# Targeting HIV-1 Reverse Transcriptase Using a Fragment-Based Approach

**DOI:** 10.3390/molecules28073103

**Published:** 2023-03-30

**Authors:** Mahta Mansouri, Shawn Rumrill, Shane Dawson, Adam Johnson, Jo-Anne Pinson, Menachem J. Gunzburg, Catherine F. Latham, Nicholas Barlow, George W. Mbogo, Paula Ellenberg, Stephen J. Headey, Nicolas Sluis-Cremer, David Tyssen, Joseph D. Bauman, Francesc X. Ruiz, Eddy Arnold, David K. Chalmers, Gilda Tachedjian

**Affiliations:** 1 Medicinal Chemistry, Monash Institute of Pharmaceutical Sciences, Monash University, Parkville, VIC 3052, Australia; 2 Center for Advanced Biotechnology and Medicine, Department of Chemistry and Chemical Biology, Rutgers University, Piscataway, NJ 08854, USA; 3 Retroviral Biology and Antivirals Laboratory, Disease Elimination Program, Life Sciences Discipline, Burnet Institute, Melbourne, VIC 3004, Australia; 4 Division of Infectious Diseases, Department of Medicine, University of Pittsburgh School of Medicine, Pittsburgh, PA 15261, USA; 5 Department of Microbiology, Monash University, Clayton, VIC 3168, Australia; 6 Department of Microbiology and Immunology, Peter Doherty Institute for Infection and Immunity, University of Melbourne, Melbourne, VIC 3000, Australia

**Keywords:** HIV-1, reverse transcriptase, fragment-based drug design, drug discovery, non-nucleoside reverse transcriptase inhibitors (NNRTIs)

## Abstract

Human immunodeficiency virus type I (HIV-1) is a retrovirus that infects cells of the host’s immune system leading to acquired immunodeficiency syndrome and potentially death. Although treatments are available to prevent its progression, HIV-1 remains a major burden on health resources worldwide. Continued emergence of drug-resistance mutations drives the need for novel drugs that can inhibit HIV-1 replication through new pathways. The viral protein reverse transcriptase (RT) plays a fundamental role in the HIV-1 replication cycle, and multiple approved medications target this enzyme. In this study, fragment-based drug discovery was used to optimize a previously identified hit fragment (compound **B-1**), which bound RT at a novel site. Three series of compounds were synthesized and evaluated for their HIV-1 RT binding and inhibition. These series were designed to investigate different vectors around the initial hit in an attempt to improve inhibitory activity against RT. Our results show that the 4-position of the core scaffold is important for binding of the fragment to RT, and a lead compound with a cyclopropyl substitution was selected and further investigated. Requirements for binding to the NNRTI-binding pocket (NNIBP) and a novel adjacent site were investigated, with lead compound **27**—a minimal but efficient NNRTI—offering a starting site for the development of novel dual NNIBP-Adjacent site inhibitors.

## 1. Introduction

Human immunodeficiency virus type 1 (HIV-1) is a retrovirus that targets cells of the immune system of the human host. If untreated, HIV-1 infection leads to acquired immunodeficiency syndrome (AIDS) and eventually death. While advances in treatment have made HIV-1 a manageable chronic condition in many cases, the continued emergence of drug-resistant variants as well as drug intolerance and drug toxicity pose substantial threats to our current therapies [1]. Therefore, the proactive discovery of new and effective drug classes with distinct drug resistance profiles to existing antiretrovirals is crucial, especially for heavily treatment-experienced individuals who have limited options for HIV treatment [2].

HIV-1 reverse transcriptase (RT) is a virally encoded enzyme that transcribes the viral RNA genome into a proviral DNA precursor prior to integration into the host genome. RT is an important target for anti-HIV drugs, and nearly 50% of U.S. Food and Drug Administration approved antiretroviral therapies target this enzyme. RT inhibitors belong to two major classes: nucleoside/nucleotide reverse transcriptase inhibitors (NRTIs) and non-nucleoside reverse transcriptase inhibitors (NNRTIs) [3]. NRTIs are analogues of the natural dNTP substrate and bind to the active site of RT. NNRTIs bind to a site that is proximal but distinct from the active site and act by an allosteric inhibition mechanism, locking RT into a non-active state [3]. Although these two classes of drugs are effective for HIV-1 treatment and prevention, continued efforts to minimize drug resistance and side effects associated with lifelong antiretroviral therapy are desirable by identification of new druggable sites on RT and continued development of new compounds [4].

**Figure 1:**
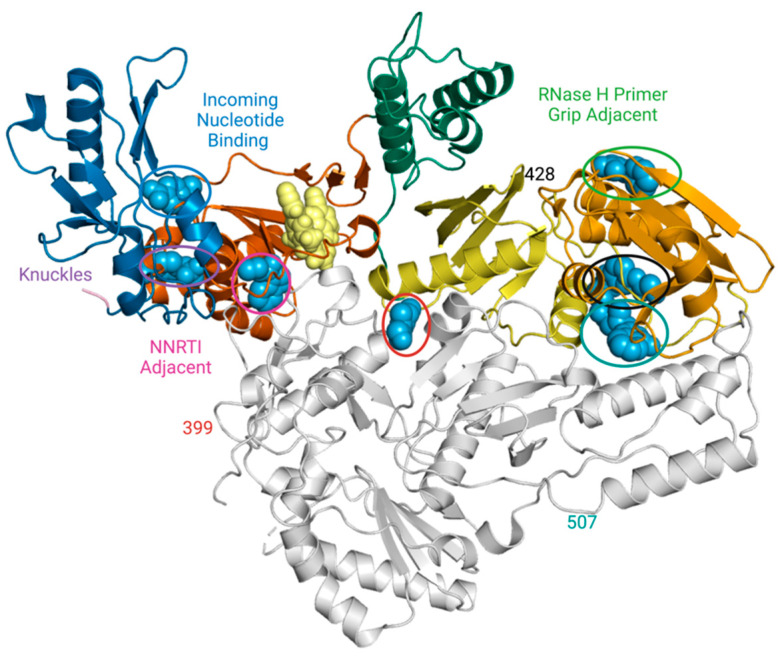
Overview of fragment hit locations in newly identified sites by Bauman et al. [7]. Legend: HIV-1 RT p66 subunit with fingers (blue), palm (red), thumb (green), connection (yellow) and RNase H (orange); p51 shown in gray; rilpivirine bound to the NNRTI-binding pocket (NNIBP) is shown as yellow spheres; bound fragments are shown as cyan spheres. Each binding site is circled and color coded according to its name (i.e., purple corresponds to Knuckles). Site, compound, and potency information in Table S1. Created with PyMOL and BioRender.com.

One relatively recent approach to discovering new druggable sites and compounds is fragment-based drug discovery (FBDD). FBDD aims to develop small molecule drugs with good pharmaceutical properties starting with chemical building blocks of drugs that are subsequently elaborated into larger more potent inhibitors [5,6]. In FBDD, libraries of low molecular weight chemical compounds, typically 300 Da or less, are screened using biophysical methods for binding to the protein target, with the notion that smaller molecules can make efficient binding interactions [7]. In 2013, Bauman et al. [7] reported an X-ray crystallographic fragment screen targeted towards HIV-1 RT. The screening library consisted of 775 commercially available compounds that were grouped into 143 cocktails. The library was screened against crystals of a crystallographically optimized HIV-1 RT variant (RT52A) in complex with the NNRTI inhibitor rilpirivine (RPV). Thirty-four fragments were identified that bound to 16 different locations on the enzyme. Seven of the binding sites had not been previously observed and have been named the NNRTI Adjacent, Knuckles, Incoming Nucleotide Binding, 399, 428, 507 and RNase H Primer Grip Adjacent binding sites (Figure 1). Of the new sites, compounds bound to four sites (Knuckles, Incoming Nucleotide Binding, 507 and NNRTI Adjacent) were reported to be inhibitory against HIV-1 RT in a dual activity assay assessing DNA polymerization and RNase H activities [7]. These sites afforded exciting new opportunities for drug development.

In particular, the NNRTI Adjacent site is a potentially attractive target for the design of novel inhibitors targeting both this site and the NNRTI-binding pocket (NNIBP) since the key residues of the pocket are conserved [7]. Accordingly, the goal of this work was to follow a systematic fragment development process to elaborate the promising hit compound **B-1** in an effort to target the NNRTI Adjacent site.

The Bauman et al. study reported compound **B-1** to have an IC_50_ of 350 μM and a ligand efficiency (LE) of 0.34 kcal/mol [7] (Table S1). LE is an expression of binding energy accounting for a compound’s size, whereby 0.30 kcal/mol or greater is considered efficient (further defined below). The fragment screen showed that this compound binds to the NNRTI Adjacent site, a region neighboring the NNIBP (Figure 1), where it makes a hydrogen bond with the backbone carbonyl oxygen of isoleucine (Ile180) and hydrophobic interactions with proline and isoleucine residues (Pro140 and Ile180). The side chains of two glutamine residues (Gln161 and Gln182) reposition to accommodate the fragment, allowing the formation of a hydrogen-bonding interaction with the glutamine (Gln182) backbone amide which was proposed to be responsible for RT inhibition by this fragment (see Figure 2b in [7]).

In this study, we aimed to optimize the binding and HIV-1 RT inhibitory activity of compound **B-1** in pursuit of a novel RT drug candidate which overcomes drug resistance and minimizes side effects associated with current antiretroviral therapies. Three series of compounds were designed, synthesized and evaluated for their HIV-1 RT binding and inhibition. A derivative **27** with an alkyne extension at the 4-position yielded an IC_50_ value of 11 μM against wild-type (WT) RT and 34 μM against the K103N/Y181C mutant RT—a variant that is resistant to many NNRTIs [8].

## 2. Results

### 2.1. Compound Synthesis

**Figure 2:**
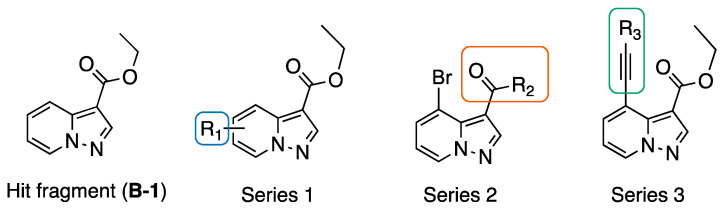
The compound **B-1** and the structural features of the three series synthesized in this study; Series 1 with changes around the core depicted in blue, Series 2 with changes to the ester depicted in orange and Series 3 with various alkyne extensions depicted in green.

Beginning with compound **B-1** as a core structure, we designed and synthesized three series of compounds (Figure 2) to identify positions that could be substituted to improve affinity for HIV-1 RT. Series 1 investigated substitution at positions 4–7 around the pyrazolopyridine ring. Series 2 modified the ethyl ester. Substitution at the 4-position was found to better inhibit RT (discussed in the following inhibition and binding section), which led to the synthesis of a set of 4-alkynes (Series 3).

**Scheme 1:**
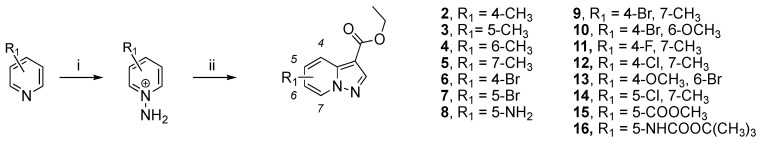
The synthesis of Series 1 analogues. **Reagents and conditions:** (i) DNPH, MeCN:H_2_O (1:1) or THF:H_2_O (1:1), 40 °C–60 °C, overnight; (ii) ethyl propiolate, K_2_CO_3_, DMF, rt, overnight.

Pyrazolopyridines with substitution on the pyridine ring (**2–16**) were prepared using the method by Kendall et al. [9,10], by *N*-amination of the substituted pyridines followed by 1,3-dipolar cycloaddition (Scheme 1). *N*-Amination of the pyridine starting materials was performed with commercially available *O-*(2,4-dinitrophenyl)hydroxylamine (DNPH) as it is more stable, easier to handle and more efficient than other reagents [11]. Acetonitrile was initially used as the solvent for this step following Kendall’s method. A method described by Legault and Charette using THF and water mixture (1:1) gave improved yields and faster reaction times [11]. Other studies speculate that the presence of water as co-solvent increases reaction speed by stabilizing the transition state [12]. This led to the inclusion of water in this reaction where maximum conversion was observed in MeCN:H_2_O (1:1) at a temperature of 60 °C. The *N*-aminopyridines were used without any purification and elaborated to pyrazolopyridines by 1,3-dipolar cycloaddition reaction with ethyl propiolate. The pyrazolopyridines were obtained in moderate yields of 15–55%, depending on the reactivity of the substituted pyridine starting materials. It was found that pyridines with electron-withdrawing substituents were not reactive enough to undergo the *N*-amination reaction. Notably, two regioisomers were formed when 3-substituted pyridines were used as the starting material, providing mixtures of 4- and 6-substituted pyrazolo[1,5-*a*]pyridines. In these cases, the two regioisomers were separated by column chromatography and distinguished using ^1^H NMR.

**Scheme 2:**
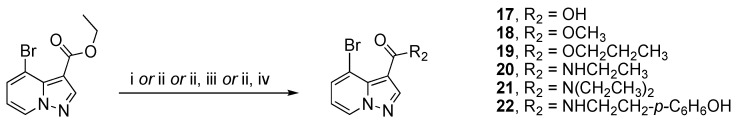
The synthesis of Series 2 analogues. **Reagents and conditions:** (i) K_2_HPO_4_, MeOH, 80 °C, overnight; (ii) LiOH. H_2_O, THF:MeOH:H_2_O (4:1:1), rt, 5 h. (iii) H_2_SO_4_, propanol, 85 °C, overnight; (iv) RNH_2_, HBTU, DIPEA, DMF, rt, 4 h.

A selection of amides and esters were synthesized to investigate the importance of the ethyl ester present in compound **B-1** (Scheme 2). Acid **17** was prepared by hydrolysis of ester **6**. Methyl ester **18** was prepared by transesterification of ethyl ester **6** using dipotassium phosphate and methanol. Compound **19** was made by esterification of acid **17** using sulfuric acid and propanol. Amides **20–22** were synthesized from acid **17** using standard amide coupling conditions.

**Scheme 3:**
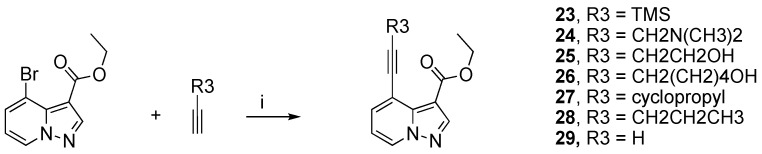
The synthesis of Series 3 analogues. **Reagents and conditions:** (i) Pd(PPh_3_)_4_, CuI, diisopropylamine, 80 °C, overnight.

Finally, a series of 4-alkynyl analogues (**23–29**) was prepared using a modification of the method by Schilz [13], using tetrakis(triphenylphosphine)palladium(0) as the catalyst, copper iodide as the co-catalyst and diisopropylamine as the base and solvent (Scheme 3). Couplings were performed at 80 °C with complete conversion observed overnight.

### 2.2. Inhibition and Binding Affinity of Pyrazolo[1,5-a]pyridine Analogues

The inhibitory activity of all compounds was evaluated in vitro in assays that measure the DNA-dependent DNA polymerase (DDDP) activity of HIV-1 RT. Compounds were tested against recombinant WT and drug-resistant HIV-1 RT mutants (K103N, Y181C or K103N/Y181C). Inhibition of WT RT DDDP activity was initially evaluated by testing three concentrations of compound in two independent assays using nevirapine (an FDA approved NNRTI) as the positive control [3]. Compounds that demonstrated WT RT inhibitory activity at concentrations less than 1 mM were then subjected to a second assay to calculate the compound IC_50_ value from dose–response curves generated from at least five serial dilutions in at least two independent assays [14]. The IC_50_ value of nevirapine was determined in parallel to control for the performance of the assay. As part of the evaluation cascade, compounds with IC_50_ values lower than 200 µM against WT RT were also tested against NNRTI-resistant recombinant RT mutants, K103N, Y181C or K103N/Y181C. These RT mutations either reduce the rate of entry of NNRTIs to the binding pocket (K103N) or remove aromatic interactions that favor binding (Y181C) [3]. Initially, compound IC_50_ values were determined in an assay that quantifies DDDP activity mediated by HIV-1 RT using a radiolabeled ^33^PdTTP substrate [14] and more recently using a nonradioactive PicoGreen-based spectrophotometric assay [15]. Accordingly, we first confirmed that the nevirapine IC_50_ value for inhibiting recombinant HIV-1 RT obtained in the non-radioactive assay was similar to the assay using ^33^PdTTP. The nevirapine IC_50_ ± SEM for inhibiting WT RT using the nonradioactive DDDP assay was 0.44 ± 0.03 µM (*n* = 3), which was not significantly different compared to the IC_50_ value reported using the ^33^PdTTP assay (0.44 ± 0.08 µM, *p* = 0.7, *n* = 3, Mann–Whitney test) [14], providing confidence that IC_50_ data generated by both assays are comparable. Inhibitory activities of Series 1 and 3 are summarized in Table 1 and Table 2. The activities of Series 2, which was largely inactive, are shown in the supporting information (Table S2).

Compound binding to recombinant HIV-1 RT was measured by surface plasmon resonance (SPR). SPR is an optical biosensor detection method that involves passing an analyte (often a fragment) in a buffer solution over a gold surface chip with an immobilized protein (RT enzyme) and detecting the change in plasmon resonance angle which is dependent on the refractive index of the medium near the surface [16,17]. As compounds bind to the target protein, the refractive index changes proportionally to the extent of binding. This can then be used to measure the association and dissociation kinetics of binding as well as the compound’s affinity for binding [17,18,19,20]. SPR is a label-free method that does not require large amounts of protein and is often used for primary screening of fragments. Binding affinities of compounds are summarized in Table 1, Table 2 and Table S2 with SPR curves shown for compounds **2**, **6** and **27** in Figure S1.

Due to their small size, fragments bind weakly; however, they are capable of binding efficiently to the target protein on a per-atom basis. Binding efficiency can be quantified by the ligand efficiency (LE) metric [21]. LE is the free energy of binding of a ligand (ΔG) divided by the number of non-hydrogen (heavy) atoms (N) where ΔG is calculated using the equation ΔG = −RTlnK_i_ [6]. Using the RT inhibition (IC_50_), LE can be calculated using the equation LE = −1.4(logIC_50_)/N [22]. Compounds with LE values greater than 0.3 kcal/mol/HA are generally regarded as efficient binders [23,24,25]. LE values for binding to WT RT are shown in Table 1 and Table 2. Notably, nearly all of the active compounds bind efficiently (LE > 0.3 kcal/mol/HA), and compound **27** binds with LE = 0.37 kcal/mol/HA.

Table 1 shows RT inhibition and SPR dissociation constants for Series 1. The hit compound **B-1** for the NNRTI Adjacent site was found to have a lower potency when tested in the DNA-dependent DNA polymerase (DDDP) activity assay used in this study compared to data from Bauman et al. which used an assay that detects inhibition of both DNA polymerase and RNase H activity [6]. Substitution at some sites on the pyridine greatly reduced inhibition. However, incorporation of bromo or methyl substituents at the 4-position improved inhibition of the WT enzyme, and this activity was somewhat maintained in the mutant strains where no significant differences were observed for the compound **6** IC_50_ value for WT RT compared to the K103N (*p* = 0.1, *n* ≥ 2) or the Y181C (*p* = 0.4, *n* ≥ 2 Mann–Whitney test) RT mutants. Notably, compounds **6** and **9** have IC_50_ values of about 150 µM, where the IC_50_ values of **5** and **6** were significantly different (*p* = 0.037, *n* = 3, Kruskal–Wallis test). These data suggest that the 4-position of the pyrazolo[1,5-*a*]pyridine core is important for inhibition of HIV-1 RT.

Series 2 compounds, which investigated modifications of the ethyl ester present in compound **B-1**, were nearly all inactive (Table S2) in the RT inhibition assay. The fact that small structural changes (e.g., changing the ethyl ester to an ethyl amide or propyl or methyl ester) in this region greatly reduced the IC_50_ values suggests that the presence of the ethyl ester is essential for RT inhibition.

The activities of the compounds in Series 3, which all contain an alkyne substituent in the 4-position, are shown in Table 2. A number of these compounds have IC_50_ values under 50 μM against recombinant WT RT as well as the K103N/Y181C NNRTI drug-resistant mutant. Notably the 4-cyclopropylalkyne **27** had an IC_50_ value of 11 μM for WT RT and was amongst the most potent compounds compared to **28** (*p* = 0.035, Kruskal–Wallis test), as well as demonstrating similar activity for inhibiting the K103N/Y181C RT mutant (*p* = 0.2, *n* ≥ 2, Mann–Whitney test). Compound **27** also demonstrated the strongest binding to both WT and mutant RT as determined by SPR (Table 2). Accordingly, this compound was selected for crystallographic studies.

### 2.3. Crystallography

**Figure 3:**
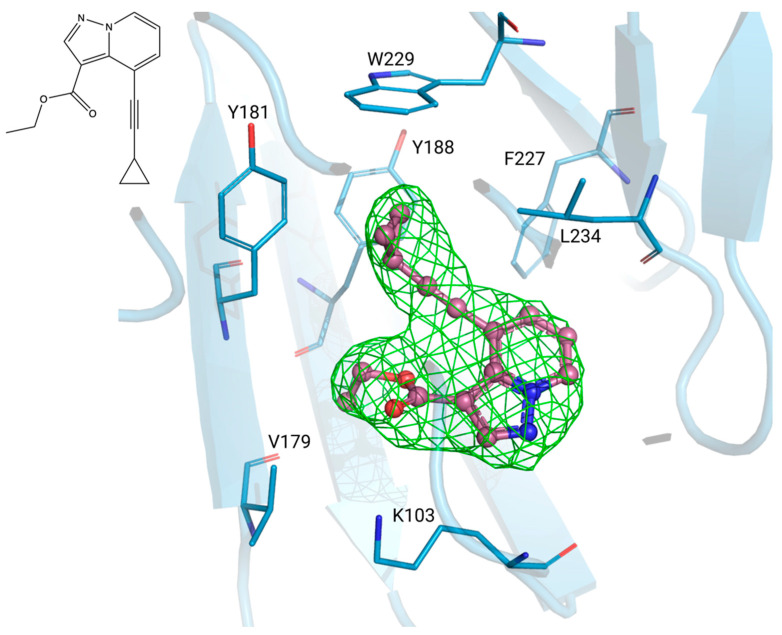
Crystal structure of HIV-1 RT in complex with the elaborated compound **27** (PDB ID 8FFX). Atomic model of **27** (magenta) bound to HIV-1 RT residues (blue) in the NNRTI-binding pocket. Pocket residues forming hydrophobic interactions shows as sticks. Polder mFo–DFc (OMIT) map density (green mesh, 3σ) of **27**. Created using PyMOL and BioRender.com.

To understand the structural basis for the improved activity of compound **27**, we determined a co-crystal structure of **27** in complex with RT52A. Initially, we attempted to co-crystallize compound **27** (anticipating its binding to the NNRTI Adjacent site) with the NNRTI rilpivirine. However, compound **27** was not observed in these experiments. Accordingly, we next solved the structure of compound **27** complexed with RT52A in the absence of an NNRTI. The structure was solved by molecular replacement using a high-resolution RT-RPV complex [26], (PDB ID 4G1Q) as the search model, and was subsequently refined to 2.42 Å resolution (Table S3). Unexpectedly, the structure showed that **27** does not bind to the NNRTI Adjacent site [7] but instead occupies the NNRTI-binding pocket (NNIBP) of RT (Figure 3). A Polder OMIT map (green mesh, Figure 3), calculated by excluding the bulk solvent around the omitted region, clearly showed the difference in electron density for **27** in the NNIBP. The cyclopropyl group of **27** is directed into the hydrophobic tunnel of the NNIBP, where it makes interactions with Y181, Y188 and W229. The N1 nitrogen of the pyrazolo[1,5-*a*]pyridine ring forms a hydrogen-bonding interaction with a water molecule mediating interaction with K103 (Figure S3). The terminal methyl of the ethyl ester group forms an additional hydrophobic interaction with V179. The much greater affinity of RPV for the NNIBP compared to **27** (0.73 nM [27] vs. 11 μM) helps explain why RPV and not **27** was observed in co-crystallization experiments of RT with **27** and RPV.

**Figure 4:**
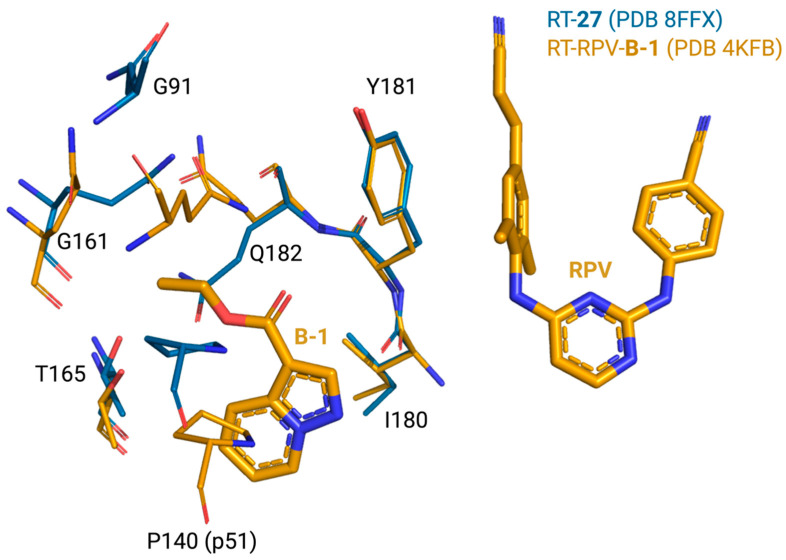
Comparison of the NNRTI Adjacent site of superposed RT-**27** (PDB ID 8FFX, blue) and RT-RPV-**B-1** (PDB ID 4KFB, orange) structures. Rilpivirine (RPV) is depicted in the neighboring NNIBP (orange). Nitrogen atoms are blue and oxygen atoms are red.

Next, we sought to understand what structural features, if any, contributed to not observing **27** in the NNRTI Adjacent site. Inspection of the NNRTI Adjacent site in the RT52A-**27** complex reveals extensive structural rearrangement that would preclude binding of **27** to the NNRTI Adjacent pocket (Figure 4). A superposition of WT RT bound to RPV with **B-1** in the NNRTI Adjacent site (PDB ID: 4KFB) onto the RT-**27** structure suggests that G161 and Q182 move downward into the NNRTI Adjacent site, where the ethyl ester group of **B-1** resides, and P140 of the p51 subunit moves upward, which would block binding of the cyclopropylethyne group **27** in the site (Figure 4). Moreover, a favorable water bridge interaction is observed between the carbonyl of **27** and K103 (Figure S3). Effectively, the NNIBP acts more as a “sink” likely providing a more favorable binding site for **27**, though not as favorable as for RPV as noted above.

### 2.4. Combination Assay

Compound **27** demonstrated a nonsignificant 3.0-fold decrease in susceptibility to the K103N/Y181C NNRTI-resistant mutant compared to WT RT (Table 2). Re-testing of compound **B-1** in the non-radioactive PicoGreen RT assay revealed IC_50_ values of 96 µM and 208 µM against WT RT and the K103N/Y181C NNRTI drug-resistant RT mutant (*p* = 0.1, *n* = 3, Mann–Whitney test), respectively (Figure S2). Thus, compound **B-1** shows a nonsignificant decrease in susceptibility to the NNRTI drug-resistant mutant compared to WT RT. The PicoGreen RT assay uses a different DNA template compared to the radioactive assay, where the latter employs activated calf thymus DNA which may account for the difference observed for RT inhibitory activity of compound **B**-**1** in the two assays. Taking together the X-ray crystallographic data and their ability to retain inhibitory activity against the NNRTI-resistant mutants, we hypothesized that compounds **B-1** and **27** bind to more than one site on the HIV-1 RT.

To investigate this further, we determined whether compound **B-1** and **27** in combination with the NNRTI, nevirapine, demonstrate additive, antagonistic or synergistic inhibition of HIV-1 RT. If compounds bind solely to the NNIBP, we would expect to see additive inhibition of RT activity in combination with nevirapine [8]. Combination assays were performed using the non-radioactive DDDP assay with drug combinations tested at fixed ratios. As a control for additivity, we tested the NNRTIs, nevirapine and doravirine (DOR) in combination [8]. While the combination of NVP and DOR was additive, the combinations of NVP and **B**-**1** and NVP and **27** displayed moderate antagonism for inhibiting HIV-1 RT DDDP activity (Table 3). These data indicate that **B**-**1** and **27** do not behave similarly to classical NNRTIs and that they may also bind to sites that are distinct from the NNRTI-binding pocket. Since these compounds displayed moderate antagonism, it is likely that binding to one site may impede the ability to bind to the second site. Given the proximity of the NNIBP to the NNRTI Adjacent site, it is possible that compounds are binding to both sites; however, this would need to be independently verified in a future work.

## 3. Discussion

### 3.1. SAR

The initial fragment hit reported by Bauman et al., (ethyl pyrazolo[1,5-*a*]pyridine-3-carboxylate, **B-1**) [7], was found in their RT activity assay to have an IC_50_ value of 350 µM for the WT RT construct RT35A with a ligand efficiency of 0.34 kcal/mol/HA. In the current study, we have used a range of synthetic techniques to elaborate compound **B**-**1**. Three focused fragment libraries were designed, synthesized and characterized. Series 1 (Table 1) explored the effects of different substitutions around the pyridine ring. Compounds **2**, **5**, **6**, **9** and **10** showed markedly improved binding and/or inhibitory activities, highlighting the importance of the 4-position. Series 2 (Table S2) investigated changes to the ethyl ester group. In this case, all changes to the ester (change in alkyl chain size, conversion to an amide) abolished inhibition and binding activities. This suggests that the ester group makes close contact and strong interactions with its binding site. Following on from the improved activities of 4-substituted compounds, Series 3 was designed. The 4-bromo substituted compound, **6**, was used as a starting material to prepare a series of alkynes through Sonogashira couplings (Table 2). Compounds **26**, **27**, **28** and **29** from this series demonstrated further improvements in activity, all having IC_50_ values less than 100 μM, which is a >20-fold improvement on parent compound **B**-**1**, which had an IC_50_ >1000 μM in the radioactive RT assay. Notably, compound **27** was among the most potent in both the RT inhibition and the SPR assay with an IC_50_ of 11 ± 1 μM and 34 ± 8 μM (*p* = 0.2) and *K*_D_ of 79 ± 7 μM and 187 ± 15 μM against WT HIV-1 RT and the K103N/Y181C double mutant, respectively. The 2–3-fold decrease in binding and non-significant change in RT inhibition mediated by **27** is encouraging, considering the small size of the compound. Compound **27** binds efficiently to WT RT with an LE of 0.40 kcal/mol/HA. To establish that the fragment series represented unique compounds, a Tanimoto similarity search was conducted against 1524 HIV-1 RT inhibitors recorded in the Binding Database (www.bindingdb.org, accessed on 9 November 2021). Of the 1524 RT inhibitors, no compound had a Tanimoto similarity score greater than 0.63. Compound **27** was selected as the lead compound to be further investigated, with crystallography and combination studies.

### 3.2. Structural Analysis

The X-ray structure of Bauman et al. demonstrated the ability of compound **B**-**1** to bind in the NNRTI Adjacent site when the drug RPV is present in the NNIBP, while displaying significant inhibition of RT polymerase activity in the absence of RPV (Table S1) [7]. However, contrary to our initial expectations, the structure of the elaborated compound **27** in complex with RT52A (in the absence of RPV) showed that the compound bound in the NNIBP (Figure 3). We surmise that, with the exception of RPV or NNRTIs with central moieties extending towards the solvent, e.g., K07-15, PDB ID 7KWU [28], NNRTIs do not induce opening of the NNRTI Adjacent site, which stays occluded and prevents binding of ligands. Of note, mining of the literature revealed that the similar shaped fragment, bromoindanone, was found as a putative NNRTI hit in an SPR fragment screening campaign targeting HIV-1 RT [29].

**Figure 5:**
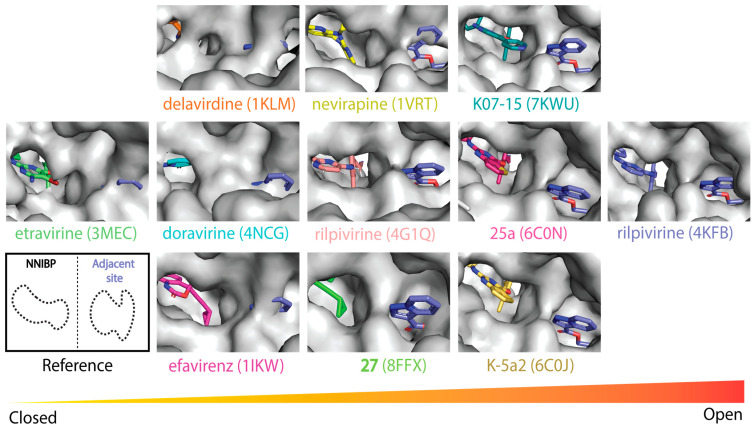
Surface representations of the NNIBP and NNRTI Adjacent sites with approved NNRTIs and investigational inhibitors. The compactness of the NNRTI Adjacent site in structures decreases from left to right (closed to open). NNRTIs and their protein bound structures are indicated in each panel, with their PDB ID in parenthesis. Lead compound **B-1** (purple) is superimposed in the NNRTI Adjacent site (right). Compound in the NNRTI site are shown in a range of colors. Nitrogen atoms are blue and oxygen atoms are red.

To understand the nature of the NNRTI Adjacent site and whether it is open to compound binding in other structures, we compared the site when either FDA approved NNRTIs or other inhibitors were bound to the NNIBP of RT. Figure 5 shows the NNIBP (left) and the NNRTI Adjacent site (right) in various inhibitor-bound structures. Each inhibitor and the associated PDB are indicated underneath each panel. Compound **B**-**1** is superimposed into the NNRTI Adjacent site in each structure, except for PDB ID 4KFB, which captures a bound state of RT52A/RPV/**1**.

The surface representations help to visualize that the NNRTI Adjacent site remains closed in most of the NNRTI-bound structures and unavailable for compounds to bind. In contrast, inhibitors **K07-15**, **25a** and **K-5a2** (Figure S5) revealed an NNRTI Adjacent site very similar to that observed in the RPV-bound structure (Figure 5), in which the NNRTI Adjacent site is open. To further investigate this observation, a pocket analysis was performed using the PyVOL extension in PyMOL (Table 4) [30].

**Figure 6:**
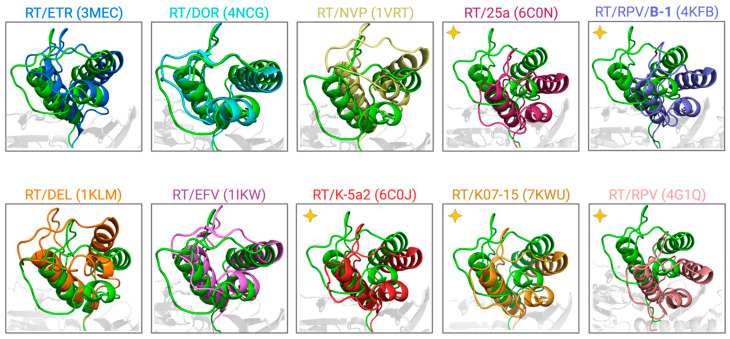
Thumb subdomain position of RT in complex with approved or investigational NNRTIs. The structure of RT bound to **27** (PDB: 8FFX) is superimposed in green in each panel. Stars indicate complexes with measurable NNRTI Adjacent Site pocket volume (Table 4). Definitions: ETR—etravirine; DOR—doravirine; NVP—nevirapine; DEL—delavirdine; EFV—efavirenz; RPV—rilpivirine. Created with PyMOL and BioRender.com.

The pocket volume analysis confirmed the surface visual analysis. Compared to RPV, K07-15, 25a or K-5a2, other approved NNRTIs complexed with HIV-1 RT have smaller NNIBP volumes and no measurable pocket volume for the NNRTI Adjacent site. Additional analysis shows that the NNIBP and the Adjacent site pocket volumes correlate with the position of the thumb in RT, which is locked into a hyperextended orientation by NNRTIs and not conducive to reverse transcription. In general, structures of RT/NNRTI with smaller NNRTI pocket volumes have lesser hyperextension in the thumb region (e.g., nevirapine, NVP; etravirine, ETR; efavirenz, EFV). However, RPV and K07-15, 25a and K-5a2, the only compounds with measurable volume for the NNRTI Adjacent site and NNIBP pocket volumes larger than 500 Å^3^, are accompanied by greater thumb hyperextension (Figure 6). This is consistent with further stabilization observed through NMR spectroscopy of the open conformation of RT (compared to closed in apo RT) when bound to diarylpyrimidine-based inhibitors (DAPYs), such as RPV, over EFV or NVP [31].

**Figure 7:**
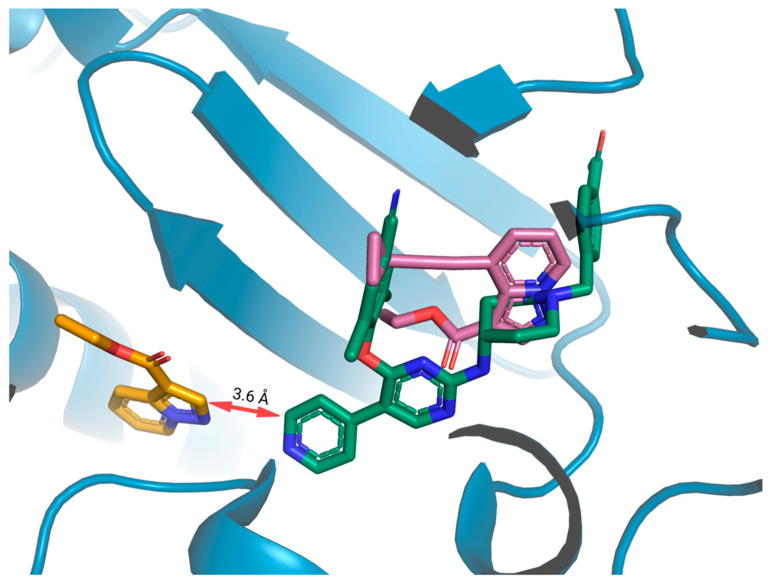
Superposition of WT RT (blue), compound **27** (magenta) and K07-15 compound (green) in the NNIBP. Compound **B-1** (orange) is shown in the NNRTI Adjacent site (PDB 4KFB). Created with PyMOL and BioRender.com. Nitrogen atoms are blue and oxygen atoms are red.

From these analyses, we conclude that there are two important structural features that may be relevant to design effective dual-targeting inhibitors binding both the NNIBP and NNRTI Adjacent site. First, an NNRTI must possess wing substituents that open the tunnel and groove channels deep enough to expand the NNIBP sufficiently to facilitate the opening of the NNRTI Adjacent pocket in the vicinity of the NNIBP solvent-exposed entrance region. Second, an aromatic extension at the 5-position of the pyrimidine ring in DAPY compounds may further open the Adjacent site and provide a bridge between the NNIBP and Adjacent site for dual-targeting inhibitor design [28,32,33]. While compound **27** in complex with RT described in this study is a relatively weak inhibitor of RT, an overlay with **K07-15** suggests the pyrazolo[1,5-*a*]pyridine ring may serve as a minimal scaffold to elaborate in designing novel dual-targeting inhibitors merged with the 5-position extension of **K07-15** (Figure 7).

In summary, we determined a crystal structure of **27** complexed with RT after elaborating it from compound **B-1**, which was previously demonstrated to target the NNRTI- Adjacent site [7]. The NNRTI Adjacent site has previously been identified as a target for enhanced NNRTI inhibition, though all leads developed had molecular weight ≥ 500 Da and borderline bioavailability properties [32,34]. The elaborated **27** acts as an NNRTI with modest inhibitory activity but retains a high ligand efficiency (0.37 kcal/mol/HA) and displays favorable bioavailability properties according to SWISSADME analysis (Figure S4) [35]. Compound **27** binding in the NNIBP is not accompanied by opening of the NNRTI Adjacent site, but crystallographic overlay with K07-15 shows a path for designing **27** derivatives to expand into the Adjacent site as a dual-targeting inhibitor with favorable pharmacokinetic properties. In this regard, the Arnold group has found additional NNRTI Adjacent fragment binders in a subsequent crystallographic fragment screening campaign that may aid in future dual-targeting inhibitor design [36].

## 4. Conclusions

In this work, using a fragment-based approach, we have developed a novel NNRTI minimal chemotype with high ligand efficiency. While the initial hit compound **B**-**1** was bound in the NNRTI Adjacent site, crystallography has revealed that binding of lead compound **27** occurs in the NNIBP. Combination experiments suggest that compounds **B**-**1** and **27** could also be binding to additional sites apart from the NNBIP that likely involve the NNRTI Adjacent site. Related to the latter, inhibitors targeting both the NNIBP and the NNRTI Adjacent site are appealing, but so far DAPY-based leads have a very large molecular weight and poor bioavailability. Structural analysis shows that compound **27**—a minimal but efficient NNRTI—offers an attractive possibility to develop a new drug class of dual NNIBP-Adjacent site inhibitors, with potential to overcome drug resistance and side effects associated with current therapeutics.

## 5. Methods and Materials

### 5.1. General Chemistry Methods

Starting materials and reagents were purchased from commercial suppliers (Sigma-Aldrich, Merck, BDH laboratories, Ajax Finechem, ChemSupply, Matrix Scientific, Alfa Aesar and Chem-Impex) and were used without further purification unless otherwise stated. Anhydrous solvents were obtained from MBraun SPS-800 Solvent purification system. TLC plates were visualized under UV illumination at 254 nm. Column chromatography was achieved using Davisil silica gel—LC60A (40–63 microns).

The ^1^H NMR, ^13^C NMR and ^19^F NMR spectra were recorded on a Bruker Advance Nanobay III 400 MHz Ultrashield Plus spectrometer at 400.13, 100.61 and 376.50 MHz, respectively. Chemical shifts (δ) are reported in parts per million (ppm), and multiplicities are reported as follows: singlet (s), doublet (d), triplet (t), quartet (q), sextet (sex), doublet of doublets (dd) and multiplet (m). All samples were dissolved in chloroform-*d* (CDCl_3_), and the residual solvent signals were used as the internal reference. The ^13^C NMR assignment of carbon environments based on APT phasing is as follows: C = quaternary carbon, CH = methine carbon, CH_2_ = methylene carbon and CH_3_ = methyl carbon.

Liquid chromatography-mass spectrometry (LC-MS) was performed using either system A or B. System A: an Agilent 6100 Series Single Quad coupled to an Agilent 1200 Series high-performance liquid chromatography (HPLC) system using a Phenomenex Luna C8 (2) 50 × 4.6 mm^2^, 5 μm column. The following buffers were used: buffer A: 0.1% formic acid in H_2_O; buffer B: 0.1% formic acid in MeCN. Samples were run at a flow rate of 0.5 mL/min for 10 min: 0–4 min 5–100% buffer B in buffer A, 4–7 min 100% buffer B, 7–9 min 100–5% buffer B in buffer A and 9–10 min 5% buffer B in buffer A. Mass spectra were acquired in positive- and negative-ion modes with a scan range of 100–1000 *m*/*z*. UV detection was carried out at 254 nm. System B: an Agilent 6120 Series Single Quad coupled to an Agilent 1260 Series HPLC using a Poroshell 120 EC-C18 50 × 3.0 mm^2^, 2.7 μm column. The following buffers were used: buffer A: 0.1% formic acid in H_2_O; buffer B: 0.1% formic acid in MeCN. Samples were run at a flow rate of 0.5 mL/min for 5 min: 0–1 min 5% buffer B in buffer A, 1–2.5 min 5–100% buffer B in buffer A, 2.5–3.8 min 100% buffer B, 3.8–4 min 100–5% buffer B in buffer A and 4–5 min 5% buffer B in buffer A. Mass spectra were acquired in positive- and negative-ion modes with a scan range of 100–1000 *m*/*z*. UV detection was carried out at 214 and 254 nm.

High resolution mass spectrometry was performed on an Agilent 6224 TOP LC/MS coupled to an Agilent 1290 Infinity. All data were acquired, and reference mass was corrected by a dual-spray electrospray ionization (ESI) source where the chromatographic separation was performed using an Agilent Zorbax SB-C18 Rapid Resolution HT 2.1 × 50 mm, 1.8 μm column using an acetonitrile gradient (5% to 100%) over 3.5 min at 0.5 mL/min. Analytical high-performance liquid chromatography (HPLC) was carried out on Agilent 1260 Infinity Analytical HPLC with a Zorbax Eclipse Plus C18 rapid resolution 4.6 × 100 mm 3.5-Micron column. PP gradient was performed using a flow rate of 1 mL/min and a gradient of 5–100% B over 9 min followed by 100% B over 1 min. LC/MSD Chemstation Rev.B.04.03 coupled with Mass Hunter Easy Access Software managed the running and processing of samples. Preparative reverse-phase HPLC was carried out using a Waters Associates liquid chromatography system (Model 600 Controller and Waters 486 Tunable Absorbance Detector) with a Phenomenex Luna C8(2) 100 Å, 10 μm, 250 × 21.2 mm column with UV detection at 254 nm. A routine slow hydrophobic run was performed using a flow rate of 10 mL/min and a gradient of 20–100% B over 35 min (solvent A: 0.1% TFA in water, solvent B: 0.1% TFA in acetonitrile).

### 5.2. General Procedure A: Cyclization of Substituted Pyridines with Ethyl Propriolate for the Formation of Substituted Ethyl Pyrazolo[1,5-a]pyridine-3-carboxylates

To a solution of substituted pyridine (1.6 mmol) in DCM (10 mL) was added *o*-(2,4-dinitrophenyl)hydroxylamine (300 mg, 1.5 mmol) at 0 °C then stirred at room temperature for 2 h. The crude material was concentrated and taken up in DMF (5 mL). Ethyl propiolate (168 μL, 1.7 mmol) and potassium carbonate (260 mg, 1.9 mmol) were added and stirred at room temperature overnight. The reaction mixture was diluted with water (100 mL), extracted with ethyl acetate (2 × 100 mL), the combined organic layers were washed with water (3 × 100 mL), brine (100 mL), dried over magnesium sulfate, filtered and concentrated in vacuo to give a gel-like brown crude product. This was purified using silica gel chromatography (hexanes:ethyl acetate, 85:15) to give the desired products.

### 5.3. Ethyl 4-Methylpyrazolo[1,5-a]pyridine-3-carboxylate (***2***) and Ethyl 6-Methylpyrazolo[1,5-a]pyridine-3-carboxylate (***4***)

3-Methylpyridine (150 mg, 1.6 mmol) was reacted using general procedure A to produce **2** as a yellow solid (55 mg, 21%) and **4** as an orange solid (26 mg, 9%).

**2: ^1^H NMR** δ 8.34 (s, 1H), 8.32 (d, *J* = 1.0 Hz, 1H), 8.05 (d, *J* = 9.0 Hz, 1H), 7.26 (dd, *J* = 9.0, 1.3 Hz, 1H), 4.36 (q, *J* = 7.1 Hz, 2H), 2.39 (d, *J* = 0.6 Hz, 3H), 1.41 (t, *J* = 7.1 Hz, 3H). **^13^C NMR** δ 163.8 (C), 144.7 (CH), 130.4 (CH), 127.4 (CH), 124.0 (C), 118.7 (CH), 177.4 (C), 60.0 (CH_2_), 18.2 (CH_3_), 14.7 (CH_3_). **HR-ESMS** calcd. for C_11_H_12_N_2_O_2_ [M+H]^+^: 205.0973; found 205.0970.

**4: ^1^H NMR** δ 8.41 (s, 1H), 8.38 (d, *J* = 6.9 Hz, 1H), 7.15–7.09 (m, 1H), 6.83 (t, *J* = 7.0 Hz, 1H), 4.34 (q, *J* = 7.1 Hz, 2H), 2.83 (s, 3H), 1.40 (t, *J* = 7.1 Hz, 3H). **^13^C NMR** δ 163.4 (C), 159.6 (C), 146.5 (CH), 130.4 (C), 128.0 (CH), 127.4 (CH), 113.7 (CH), 107.7 (C), 60.2 (CH_2_), 21.7(CH_3_), 14.7 (CH_3_). **HR-ESMS** calcd. for C_11_H_12_N_2_O_2_ [M+H]^+^: 205.0972; found 205.0968.

### 5.4. Ethyl 5-Methylpyrazolo[1,5-a]pyridine-3-carboxylate (***3***)

4-Methylpyridine (150 mg, 1.6 mmol) was reacted using general procedure A to produce **3** as a brown solid (175 mg, 57%).

**^1^H NMR** δ 8.39 (d, *J* = 7.0 Hz, 1H), 8.34 (d, *J* = 7.6 Hz, 1H), 6.72 (s, 1H), 7.95–7.91 (m, 1H), 6.77 (dd, *J* = 7.1, 1.9 Hz, 1H), 4.38 (q, *J* = 7.1 Hz, 2H), 2.46 (d, *J* = 0.9 Hz, 3H), 1.41 (t, *J* = 7.1 Hz, 3H). **^13^C NMR** δ 145.3 (CH), 141.4 (C), 138.9 (C), 128.7 (CH), 117.9 (CH), 116.5 (CH), 60.3 (CH_2_), 21.7 (C), 15.0 (CH_3_). **HPLC** *t*_R_ = 5.49 min, >95% purity (254 nm). **HR-ESMS** calcd. for C_11_H_12_N_2_O_2_ [M+H]^+^: 205.0972; found 205.0968.

### 5.5. Ethyl 7-Methylpyrazolo[1,5-a]pyridine-3-carboxylate (***5***)

3-Methylpyridine (170 mg, 1.9 mmol) was reacted using general procedure A to produce **5** as an orange solid (133 mg, 36%).

**^1^H NMR** δ 8.44 (s, 1H), 8.08 (dd, *J* = 8.9, 0.5 Hz, 1H), 7.35 (dd, *J* = 8.8, 7.0 Hz, 1H), 6.81 (d, *J* = 6.9 Hz, 1H), 4.39 (q, *J* = 7.1 Hz, 2H), 2.80 (s, 3H), 1.42 (t, *J* = 7.1 Hz, 3H). **^13^C NMR** δ 164.0 (C), 144.5 (CH), 141.5 (C), 139.4 (C), 127.6 (CH), 116.9 (CH), 113.4 (CH), 104.3 (C), 60.2 (CH_2_), 18.0 (CH_3_), 14.7 (CH_3_). **HPLC** *t*_R_ = 3.39 min, > 95% purity (254 nm). **HR-ESMS** calcd. for C_11_H_12_N_2_O_2_ [M+H]^+^: 205.0972; found 205.0970.

### 5.6. Ethyl 5-Aminopyrazolo[1,5-a]pyridine-3-carboxylate (***8***)

To a solution of ethyl 5-((*tert*-butoxycarbonyl)amino)pyrazolo[1,5-*a*]pyridine-3-carboxylate (95 mg, 0.31 mmol) in DCM (3 mL) was added dropwise TFA (480 μL, 6.2 mmol) and then stirred at room temperature for 21 h. Crude mixture was concentrated to give **8** as a tan solid (100 mg, quantitative 100%).

**^1^H NMR** δ 8.46 (d, *J* = 7.4 Hz, 1H), 8.28 (s, 1H), 7.23 (d, *J* = 2.2 Hz, 1H), 6.46 (dd, *J* = 7.4, 2.3 Hz, 1H), 6.50–6.33 (bs, 2H), 4.35 (q, *J* = 7.1 Hz, 2H), 1.39 (t, *J* = 7.1 Hz, 3H). **HPLC** *t*_R_ = 2.92 min, >95% purity (254 nm). **HR-ESMS** calcd. for C_10_H_11_N_3_O_2_ [M+H]^+^: 206.0924; found 206.0923.

### 5.7. 3-Ethyl 5-Methylpyrazolo[1,5-a]pyridine-3,5-dicarboxylate (***15***)

Methyl isonicotinate (220 mg, 1.6 mmol) was reacted using general procedure A to produce **15** as an orange solid (89 mg, 24%).

**^1^H NMR** δ 8.86 (dd, *J* = 1.9, 0.9 Hz, 1H), 8.55 (dd, *J* = 7.2, 0.9 Hz, 1H), 8.47 (s, 1H), 7.52 (dd, *J* = 7.2, 1.9 Hz, 1H), 4.42 (q, *J* = 7.1 Hz, 2H), 3.99 (s, 3H), 1.44 (t, *J* = 7.1 Hz, 3H). **^13^C NMR** δ 165.2 (C), 163.1 (C), 145.7 (CH), 140.0 (C), 129.2 (CH), 128.8 (C), 121.8 (CH), 113.0 (CH), 106.9 (C), 60.5 (CH_2_), 53.0 (CH_3_), 14.8 (CH_3_). **HPLC** *t*_R_ = 3.37 min, >95% purity (254 nm). **HR-ESMS** calcd. for C_11_H_12_N_2_O_2_ [M+H]^+^: 249.0870; found 249.0869.

### 5.8. Ethyl 5-((Tert-butoxycarbonyl)amino)pyrazolo[1,5-a]pyridine-3-carboxylate (***16***)

*tert*-Butyl pyridin-4-ylcarbamate (310 mg, 1.6 mmol) was reacted using general procedure A to produce **16** as a yellow solid (110 mg, 24%).

**^1^H NMR** δ 8.39 (d, *J* = 7.6 Hz, 1H), 8.32 (s, 1H), 7.98 (d, *J* = 2.1 Hz, 1H), 7.33–7.28 (m, 1H), 6.93 (s, 1H), 4.36 (q, *J* = 7.1 Hz, 2H), 1.54 (s, 9H), 1.40 (t, *J* = 7.1 Hz, 3H). **^13^C NMR** δ 163.7 (C), 152.1 (C), 145.7 (CH), 141.8 (C), 138.8 (C), 129.6 (CH), 107.3 (CH), 104.0 (CH), 103.0 (C), 82.0 (C), 60.0 (CH_2_), 28.4 (CH_3_), 14.7 (CH_3_). **LCMS** (*m*/*z*) 305.9 [M+H]^+^, *t*_R_ = 5.94 min, >95% purity (254 nm).

### 5.9. General Procedure B: Cyclization of Substituted Pyridine with Ethyl Propriolate for the Formation of Substituted Ethyl Pyrazolo[1,5-a]pyridine-3-carboxylates

*O*-(2,4-Dinitrophenyl)hydroxylamine (1.0 eq.) was dissolved in THF: H_2_O or MeCN:H_2_O (1:1, 20 mL), substituted pyridine (1.05 eq.) was added and stirred at 50–60 °C for 18 h. The solvent was removed in vacuo, crude material was taken up in anhydrous DMF (15 mL), and then potassium carbonate (1.5 eq.) and ethyl propiolate (1.1 eq.) were added and then stirred for 20 h. The reaction mixture was diluted with water (100 mL), extracted with ethyl acetate (2 × 100 mL); the combined organic layers were washed with water (3 × 100 mL), brine (100 mL), dried over magnesium sulphate, filtered and concentrated in vacuo. The crude material was purified using silica gel chromatography (hexanes:ethyl acetate, 85:15) to give the desired products.

### 5.10. Ethyl 6-Bromopyrazolo[1,5-a]pyridine-3-carboxylate (***7***) and Ethyl 4-Bromopyrazolo[1,5-a]pyridine-3-carboxylate (***6***)

*o*-(2,4-Dinitrophenyl)hydroxylamine (2.0 g, 10 mmol), 3-bromopyridine (1.4 g, 9.1 mmol), potassium carbonate (1.9 g, 14 mmol) and ethyl propiolate (0.99 g, 10 mmol) were reacted using general procedure B to give the title compounds: **7** as a white powder (0.45 g, 18%) and **6** as a yellow solid (0.88 mg, 36%).

**6: ^1^H NMR** δ 8.52 (dd, *J* = 6.9, 0.9 Hz, 1H), 8.43 (s, 1H), 7.64 (dd, *J* = 7.4, 0.9 Hz, 1H), 6.80 (t, *J* = 7.2 Hz, 1H), 4.38 (q, *J* = 7.1 Hz, 2H), 1.41 (t, *J* = 7.1 Hz, 3H). **^13^C NMR** δ 162.4 (C), 146.5 (CH), 138.8 (C), 132.2 (CH), 129.0 (CH), 113.5 (CH), 111.6 (C), 106.3 (C), 60.7 (CH_2_), 14.5 (CH_3_). **HPLC** *t*_R_ = 5.50 min, >95% purity (254 nm). **HR-ESMS** calcd. for C_10_H_9_O_2_N_2_Br [M+H]^+^: 268.9920; found 268.9925.

**7: ^1^H NMR** δ 8.67 (dd, *J* = 1.7, 0.8 Hz, 1H), 8.36 (s, 1H), 8.06 (dd, *J* = 9.4, 0.8 Hz, 1H), 7.47 (dd, *J* = 9.4, 1.7 Hz, 1H), 4.38 (q, *J* = 7.1 Hz, 2H), 1.41 (t, *J* = 7.1 Hz, 3H). **^13^C NMR** δ 163.2 (C), 145. (CH), 139.5 (C), 130.8 (CH), 129.7 (CH), 119.7 (CH), 108.3 (C), 104.9 (C), 60.4 (CH_2_), 14.6 (CH_3_). **HPLC** *t*_R_ = 5.94 min, >98% purity (254 nm). **HR-ESMS** calcd. for C_10_H_9_O_2_N_2_Br [M+H]^+^: 268.9920; found 268.9918.

### 5.11. Ethyl 4-Bromo-7-methylpyrazolo[1,5-a]pyridine-3-carboxylate (***9***)

*o*-(2,4-Dinitrophenyl)hydroxylamine (0.10 g, 0.51 mmol), 5-bromo-2-methylpyridine (80 mg, 0.47 mmol), potassium carbonate (96 mg, 0.70 mmol) and ethyl propiolate (50 mg, 0.51 mmol) were reacted using general procedure B to produce **9** as an off-white solid (12 mg, 9%).

**^1^H NMR** δ 8.40 (s, 1H), 8.26 (d, *J* = 1.6 Hz, 1H), 6.93 (d, *J* = 1.0 Hz, 1H), 4.39 (q, *J* = 7.1 Hz, 2H), 2.77 (s, 3H), 1.42 (t, *J* = 7.1 Hz, 3H). **^13^C NMR** δ 163.4 (C), 144.9 (CH), 141.7 (C), 140.2 (C), 122.3 (C), 119.3 (CH), 116.9 (CH), 104.2 (C), 60.3 (CH_2_), 17.7 (CH_3_), 14.7 (CH_3_). **HPLC** *t*_R_ = 6.48 min, >98% purity (254 nm). **HR-ESMS** calcd. for C_11_H_11_O_2_N_2_Br [M+H]^+^: 283.0077; found 283.0086.

### 5.12. Ethyl 6-Bromo-4-methoxypyrazolo[1,5-a]pyridine-3-carboxylate (***13***) and Ethyl 4-Bromo-6-methoxypyrazolo[1,5-a]pyridine-3-carboxylate (***10***)

*o*-(2,4-Dinitrophenyl)hydroxylamine (99 mg, 0.50 mmol), 3-bromo-5-methoxypyridine (85 mg, 0.45 mmol), potassium carbonate (93 mg, 0.68 mmol) and ethyl propiolate (50 mg, 0.50 mmol) were reacted using general procedure B to give the title compounds: **10** as a white powder (14 mg, 7%) and **13** as a pale pink solid (40 mg, 30%).

**10: ^1^H NMR** δ 8.33 (s, 1H), 8.10 (d, *J* = 2.1 Hz), 7.47 (d, *J* = 2.1 Hz), 4.36 (q, *J* = 7.1 Hz), 3.85 (s, 3H), 1.39 (t, *J* = 7.1 Hz). **^13^C NMR** δ 162.4 (C), 149.5 (C), 145.8 (CH), 145.8 (C), 134.5 (C), 126.8 (CH), 111.7 (CH), 111.3 (C), 60.6 (CH_2_), 56.6 (CH_3_), 14.7 (CH_3_). **HPLC** *t*_R_ = 5.93 min, >99% purity (254 nm). **HR-ESMS** calcd. for C_11_H_11_O_3_N_2_Br [M+H]^+^: 299.0026; found 299.0030.

**13: ^1^H NMR** δ 8.31 (s, 2H), 6.72 (s, 1H), 4.34 (q, *J* = 7.1 Hz, 2H), 4.00 (s, 3H), 1.38 (t, *J* = 7.1 Hz, 3H). **^13^C NMR** δ 162.3 (C), 151.7 (C), 145.6 (CH), 132.7 (C), 122.9 (CH), 108.2 (CH), 108.0 (C), 106.4 (C), 60.5 (CH_2_), 56.5 (CH_3_), 14.5 (CH_3_). **HPLC** *t*_R_ = 5.71 min, >94% purity (254 nm). **HR-ESMS** calcd. for C_11_H_11_O_3_N_2_Br [M+H]^+^: 299.0026; found 299.0032.

### 5.13. Ethyl 4-Fluoro-7-methylpyrazolo[1,5-a]pyridine-3-carboxylate (***11***)

*o*-(2,4-Dinitrophenyl)hydroxylamine (99 mg, 0.49 mmol), 5-fluoro-2-methylpyridine (50 mg, 0.45 mmol), potassium carbonate (93 mg, 0.67 mmol) and ethyl propiolate (49 mg, 0.49 mmol) were reacted using general procedure B to produce **11** as a pale yellow solid (40 mg, 40%).

**^1^H NMR** δ 8.44 (s, 1H), 7.03 (dd, *J* = 10.2, 7.9 Hz, 1H), 6.70 (ddd, *J* = 7.8, 4.5, 0.5 Hz, 1H), 4.36 (q, *J* = 7.1 Hz, 2H), 2.73 (s, 3H), 1.39 (t, *J* = 7.1 Hz, 3H). **^13^C NMR** δ 162.6 (C), δ 152.4 (d, *J* = 254.2 Hz, C), 145.2 (CH), 135.8 (d, *J* = 5.3 Hz, C), 132.6 (d, *J* = 32.1 Hz, C), 111.8 (d, *J* = 6.2 Hz, CH), 111.4 (d, *J* = 18.8 Hz, CH), 105.2 (d, *J* = 5.6 Hz, C), 60.5 (CH_2_), 17.7 (CH_3_), 14.5 (CH_3_). **HPLC** *t*_R_ = 5.52 min, >99% purity (254 nm). **HR-ESMS** calcd. for C_11_H_11_O_2_N_2_F [M+H]^+^: 223.0877; found 223.0888.

### 5.14. Ethyl 4-Chloro-7-methylpyrazolo[1,5-a]pyridine-3-carboxylate (***12***)

*o*-(2,4-Dinitrophenyl)hydroxylamine (0.10 g, 0.52 mmol), 5-chloro-2-methylpyridine (60 mg, 0.47 mmol), potassium carbonate (98 mg, 0.71 mmol) and ethyl propiolate (51 mg, 0.52 mmol) were added were reacted using general procedure B to produce **12** as an off-white powder (62 mg, 55%).

**^1^H NMR** δ 8.44 (s, 1H), 7.34 (d, *J* = 7.6 Hz, 1H), 6.72 (d, *J* = 7.6 Hz, 1H), 4.37 (q, *J* = 7.1 Hz, 2H), 2.74 (s, 3H), 1.39 (t, *J* = 7.1 Hz, 3H). **^13^C NMR** δ 162.6 (C), 145.9 (CH), 138.4 (C), 138.0 (C), 128.2 (CH), 122.5 (C), 112.7 (CH), 106.1 (C), 60.6 (CH_2_), 18.1 (CH_3_), 14.5 (CH_3_). **HPLC** *t*_R_ = 5.92 min, >94% purity (254 nm). **HR-ESMS** calcd. for C_11_H_11_O_2_N_2_Cl [M+H]^+^: 239.0582; found 239.0589.

### 5.15. Ethyl 5-Chloro-7-methylpyrazolo[1,5-a]pyridine-3-carboxylate (***14***)

*O*-(2,4-Dinitrophenyl)hydroxylamine (0.10 g, 0.52 mmol), 4-chloro-2-methylpyridine (60 mg, 0.47 mmol), potassium carbonate (98 mg, 0.71 mmol) and ethyl propiolate (51 mg, 0.52 mmol) were reacted using general procedure B to produce **14** as an off-white solid (15 mg, 13%).

**^1^H NMR** δ 8.41 (s, 1H), 8.06 (d, *J* = 1.8 Hz, 1H), 6.79 (d, *J* = 1.1 Hz, 1H), 4.38 (q, *J* = 7.1 Hz, 2H), 2.77 (s, 3H), 1.41 (t, *J* = 7.1 Hz, 3H). **^13^C NMR** δ 163.4 (C), 145.1 (CH), 141.4 (C), 140.3 (C), 134.5 (C), 115.8 (CH), 114.5 (CH), 104.3 (C), 60.3 (CH_2_), 17.8 (CH_3_), 14.7 (CH_3_). **HPLC** *t*_R_ = 6.33 min, >98% purity (254 nm). **HR-ESMS** calcd. for C_11_H_11_O_2_N_2_Cl [M+H]^+^: 239.0582; found 239.0588.

### 5.16. 4-Bromopyrazolo[1,5-a]pyridine-3-carboxylic Acid (***17***)

Ethyl 4-bromopyrazolo[1,5-*a*]pyridine-3-carboxylate (30 mg, 0.13 mmol) was dissolved in a solution of THF:MeOH:H2O (4:1:1, 2 mL). Lithium hydroxide monohydrate (71 mg, 1.7 mmol) was added and stirred at room temperature for 24 h. Additional lithium hydroxide monohydrate was added (54 mg, 1.3 mmol) and stirred for an additional 8 h. Solvent was removed in vacuo, dissolved in ethyl acetate (10 mL) and acidified with HCl (1 M, 10 mL). The organic layer was then washed with brine, dried over magnesium sulphate, filtered and concentrated in vacuo to give **17** as a pale-yellow powder (30 mg, 96%).

**^1^H NMR** (400 MHz, (CD_3_)_2_SO) δ 12.47 (bs, 1H, OH), 8.89 (dd, *J* = 6.9, 0.9 Hz, 1H, H7), 8.45 (s, 1H, H2), 7.82 (dd, *J* = 7.5, 0.8 Hz, 1H, H5), 7.02 (t, *J* = 7.2 Hz, 1H, H6). **^13^C NMR** (100 MHz, (CD_3_)_2_SO) δ 153.2 (C), 136.2 (CH), 127.9 (C), 122.7 (CH), 120.0 (CH), 104.5 (CH), 100.3 (C), 96.5 (C). **HPLC** *t*_R_ = 3.80 min, >95% purity (254 nm). **HR-ESMS** calcd. for C_8_H_5_O_2_N_2_Br [M+H]^+^: 242.9587; found 242.9592.

### 5.17. Methyl 4-Bromopyrazolo[1,5-a]pyridine-3-carboxylate (***18***)

Ethyl 4-bromopyrazolo[1,5-*a*]pyridine-3-carboxylate (50 mg, 0.19 mmol) and dipotassium phosphate (8.0 mg, 0.05 mmol) were dissolved in methanol (3 mL) and then stirred at 80 °C for 24 h. Additional dipotassium phosphate (10 mg, 0.06 mmol) was added and stirred at 80 °C for another 24 h. The reaction mix was diluted with water (20 mL), extracted twice with ethyl acetate (2 × 20 mL) and then combined organic layers were washed with water (2 × 20 mL) and brine, dried over magnesium sulphate, filtered and concentrated in vacuo. The crude material was purified using preparative reverse-phase HPLC to give **18** as a white powder (14 mg, 30%).

**^1^H NMR** (400 MHz, CDCl_3_) δ 8.53 (dd, *J* = 6.9, 1.0 Hz, 1H, H7), 8.43 (s, 1H, H2), 7.65 (dd, *J* = 7.5, 0.9 Hz, 1H, H5), 6.81 (t, *J* = 7.2 Hz, 1H, H6), 3.91 (s, 3H, H1’). **^13^C NMR** (100 MHz, CDCl_3_) δ 162.8 (C), 146.5 (CH), 138.9 (C), 132.3 (CH), 129.1 (CH), 113.62 (CH), 111.6 (C), 105.9 (C), 51.8 (CH_3_). **HPLC** *t*_R_ = 4.91 min, >98% purity (254 nm). **HR-ESMS** calcd. for C_9_H_7_O_2_N_2_Br [M+H]^+^: 256.9744; found 256.9747.

### 5.18. Propyl 4-bromopyrazolo[1,5-a]pyridine-3-carboxylate (***19***)

4-Bromopyrazolo[1,5-*a*]pyridine-3-carboxylic acid was dissolved in propanol (3 mL) with concentrated sulfuric acid (40 μL), and the resulting mixture was stirred at 90 °C for 24 h. Additional concentrated sulfuric acid (50 μL) was added and stirred at 90 °C for another 24 h. The mixture was cooled and neutralized with NaOH (1 M) and washed twice with ethyl acetate (2 × 20 mL). The combined organic layers were then washed with brine (20 mL), dried over magnesium sulphate, filtered and concentrated in vacuo. The crude material was purified using preparative reverse-phase HPLC to give **19** as a white powder (21 mg, 36%).

**^1^H NMR** (400 MHz, CDCl_3_) δ 8.52 (dd, *J* = 6.9, 1.0 Hz, 1H, H7), 8.43 (s, 1H, H2), 7.64 (dd, *J* = 7.4, 1.0 Hz, 1H, H5), 6.80 (t, *J* = 7.2 Hz, 1H, H6), 4.28 (t, *J* = 6.8 Hz, 2H, H1’), 1.86–1.76 (sex, *J* = 7.4 Hz, 2H, H2’), 1.03 (t, *J* = 7.4 Hz, 3H, H3’). ^**13**^**C NMR** (100 MHz, CDCl_3_) δ 162.5 (C), 146.5 (CH), 146.5 (C), 132.2 (CH), 129.0 (CH), 113.5 (CH), 111.7 (C), 106.4 (C), 66.4 (CH_2_), 22.3 (CH_2_), 10.7 (CH_3_). **HPLC** *t*_R_ = 6.01 min, >98% purity (254 nm). **HR-ESMS** calcd. for C_11_H_11_O_2_N_2_Br [M+H]^+^: 283.0077; found 283.0082.

### 5.19. 4-Bromo-N-ethylpyrazolo[1,5-a]pyridine-3-carboxamide (***20***)

4-Bromopyrazolo[1,5-*a*]pyridine-3-carboxylic acid (13 mg, 0.05 mmol) was dissolved in DMF (1 mL). HBTU (2 eq.), DIPEA (4 eq.), and 70% aqueous ethylamine (4.7 mg, 0.10 mmol) were added and the solution was stirred at room temperature for 2–4 h. The crude solution was added dropwise to a mixture of water and saturated sodium bicarbonate (1:1, 30 mL), stirred for 1 h and then extracted into ethyl acetate (30 mL), washed with water (2 × 30 mL) and brine (20 mL), dried over magnesium sulphate, filtered and concentrated in vacuo to produce **20** with sufficient purity as a pale powder (12 mg, 86%).

**^1^H NMR** (400 MHz, CDCl_3_) δ 8.47 (d, *J* = 6.4 Hz, 1H, H7), 8.17 (s, 1H, H2), 7.50 (d, *J* = 6.9 Hz, 1H, H5), 6.74 (t, *J* = 7.2 Hz, 1H, H6), 6.04 (s, 1H, NH), 3.58–3.45 (m, 2H, H1’), 1.28 (t, *J* = 7.3 Hz, 3H, H2’). **^13^C NMR** (100 MHz, CDCl_3_) δ 163.2 (C), 142.9 (CH), 136.5 (C), 129.7 (CH), 128.5 (CH), 112.7 (CH), 111.0 (C), 101.2 (C), 35.2 (CH_2_), 14.8 (CH_3_). **HPLC** *t*_R_ = 3.76 min, >87% purity (254 nm). **HR-ESMS** calcd. for C_10_H_10_ON_3_Br [M+H]^+^: 268.0080; found 268.0085.

### 5.20. 4-Bromo-N,N-diethylpyrazolo[1,5-a]pyridine-3-carboxamide (***21***)

4-Bromopyrazolo[1,5-*a*]pyridine-3-carboxylic acid (13 mg, 0.05 mmol) was dissolved in DMF (1 mL). HBTU (2 eq.), DIPEA (4 eq.), and diethylamine (10 mg, 0.14 mmol) were added, and the solution was stirred at room temperature for 2–4 h. The crude solution was added dropwise to a mixture of water and saturated sodium bicarbonate (1:1, 30 mL), stirred for 1 h and then extracted into ethyl acetate (30 mL), washed with water (2 × 30 mL) and brine (20 mL), dried over magnesium sulphate, filtered and concentrated in vacuo to produce **21** with sufficient purity as a dark oil (15 mg, 74%).

**^1^H NMR** (400 MHz, CDCl_3_) δ 8.45 (dd, *J* = 7.0, 0.8 Hz, 1H, H7), 7.98 (s, 1H, H2), 7.41 (dd, *J* = 7.3, 0.8 Hz, 1H, H5), 6.71 (t, *J* = 7.2 Hz, 1H, H6), 3.63 (dd, *J* = 15.1, 8.6 Hz, 2H, H1’), 3.28 (dd, *J* = 14.0, 6.9 Hz, 2H, H1’’), 1.30 (t, *J* = 7.0 Hz, 3H, H2’), 1.08 (t, *J* = 7.0 Hz, 3H, H2’’). **^13^C NMR** (100 MHz, CDCl_3_) δ 164.5 (C), 155.1 (C), 140.4 (CH), 128.2 (CH), 128.1 (CH), 112.5 (CH), 111.1 (C), 104.4 (C), 43.8 (CH_2_), 39.6 (CH_2_), 14.2 (CH_3_), 12.5 (CH_3_). **LCMS** (*m*/*z*) 296.9 [M+H]^+^, *t*_R_ = 3.22 min. **HPLC** *t*_R_ = 4.83 min, >90% purity (254 nm).

### 5.21. 4-Bromo-N-(4-hydroxyphenethyl)pyrazolo[1,5-a]pyridine-3-carboxamide (***22***)

4-Bromopyrazolo[1,5-*a*]pyridine-3-carboxylic acid (17 mg, 0.07 mmol) was dissolved in DMF (1 mL). HBTU (2 eq.), DIPEA (4 eq.), and 4-(2-aminoethyl)phenol (19 mg, 0.14 mmol) were added, and the solution was stirred at room temperature for 2–4 h. The crude solution was added dropwise to a mixture of water and saturated sodium bicarbonate (1:1, 30 mL), stirred for 1 h and then extracted into ethyl acetate (30 mL), washed with water (2 × 30 mL) and brine (20 mL), dried over magnesium sulphate, filtered and concentrated in vacuo to produce **22** with sufficient purity as a pale oil (16 mg, 63%).

**^1^H NMR** (400 MHz, (CD_3_)_2_SO) δ 9.23 (bs, 1H, OH), 8.79 (dd, *J* = 6.9, 0.8 Hz, 1H, H7), 8.34 (t, *J* = 5.6 Hz, 1H, H6), 8.15 (s, 1H, H2), 7.65 (dd, *J* = 7.4, 0.8 Hz, 1H, H5), 7.05 (d, *J* = 8.5 Hz, 2H, H3’), 6.91 (t, *J* = 7.2 Hz, 1H, H6), 6.71–6.66 (m, 2H, H4’), 3.40 (dd, *J* = 14.7, 6.0 Hz, 2H, H1’), 2.73 (t, *J* = 7.5 Hz, 2H, H2’). **^13^C NMR** (100 MHz, (CD_3_)_2_SO) δ 162.2 (C), 155.9 (C), 142.1 (CH), 135.8 (C), 129.5 (CH × 2), 129.4 (CH), 129.3 (C), 128.8 (CH), 115.2 (CH × 2), 113.1 (CH), 111.2 (C), 110.4 (C), 41.2 (CH_2_), 34.2 (CH_2_). **HPLC** *t*_R_ = 4.410 min, >95% purity (254 nm). **HR-ESMS** calcd. for C_16_H_14_O_2_N_3_Br [M+H]^+^: 360.0342; found 360.0328.

### 5.22. General Procedure C: Sonogashira Reaction for the Formation of Ethyl 4-Substituted Pyrazolo[1,5-a]pyridine-3-carboxylates

Tetrakis(triphenylphosphine)palladium(0) (0.1 eq.), copper iodide (0.1 eq.) and ethyl 4-bromopyrazolo[1,5-*a*]pyridine-3-carboxylate (1 eq.) were combined, sealed and degassed. Diisopropylamine (~2 mL) and substituted alkyne (1.5–2 eq.) were added to the reaction mix, degassed and then stirred under nitrogen for 20 h at 80 °C. Crude material was taken up into ethyl acetate (20 mL), filtered through Celite™ and washed with water, and the aqueous layer was washed with ethyl acetate. The combined organic layers were washed with water (3 × 30 mL) and brine (20 mL), dried over magnesium sulphate, filtered and concentrated in vacuo to give crude product. This was purified using silica gel chromatography (hexanes:ethyl acetate, 9:1) to give the desired product.

### 5.23. Ethyl 4-((Trimethylsilyl)ethynyl)pyrazolo[1,5-a]pyridine-3-carboxylate (***23***)

Ethyl 4-bromopyrazolo[1,5-*a*]pyridine-3-carboxylate (36 mg, 0.13 mmol) and trimethylsilylacetylene (26 mg, 0.27 mmol) were reacted using general procedure C to produce **23** as a yellow oil (30 mg, 80%).

**^1^H NMR** δ 8.48 (dd, *J* = 6.9, 0.8 Hz, 1H), 8.38 (s, 1H), 7.56 (dd, *J* = 7.2, 0.9 Hz, 1H), 6.86 (t, *J* = 7.1 Hz, 1H), 4.36 (q, *J* = 7.1 Hz, 2H), 1.38 (t, *J* = 7.1 Hz, 3H), 0.29 (s, 9H). **^13^C NMR** δ 162.1 (C), 145.9 (CH), 139.2 (C), 133.9 (CH), 129.8 (CH), 115.1 (C), 113.0 (CH), 106.3 (C), 102.8 (C), 100.4 (C), 60.0 (CH_2_), 14.7 (CH_3_), 0.0 (3 × CH_3_). **HPLC** *t*_R_ = 7.27 min, >98% purity (254 nm). **HR-ESMS** calcd. for C_15_H_18_O_2_N_2_Si [M+H]^+^: 287.1210; found 287.1219.

### 5.24. Ethyl 4-(3-(Dimethylamino)prop-1-yn-1-yl)pyrazolo[1,5-a]pyridine-3-carboxylate (***24***)

Ethyl 4-bromopyrazolo[1,5-*a*]pyridine-3-carboxylate (50 mg, 0.19 mmol) and *N,N*-dimethylprop-2-yn-1-amine (46 mg, 0.56 mmol) were reacted using general procedure C to produce **24** as a brown oil (34 mg, 67%).

**^1^H NMR** δ 8.46 (dd, *J* = 6.9, 0.8 Hz, 1H), 8.38 (s, 1H), 7.52 (dd, *J* = 7.2, 0.7 Hz, 1H), 6.86 (t, *J* = 7.1 Hz, 1H), 4.33 (q, *J* = 7.1 Hz, 2H), 3.59 (s, 2H), 2.40 (s, 6H), 1.36 (t, *J* = 7.1 Hz, 3H). **^13^C NMR** δ 162.4 (C), 146.2 (CH), 139.5 (C), 133.5 (CH), 129.6 (CH), 115.3 (C), 113.2 (CH), 106.0 (C), 92.7 (C), 81.7 (C), 60.3 (CH_2_), 49.1 (CH_2_), 44.6 (2 × CH_3_), 14.7 (CH_3_). **HPLC** *t*_R_ = 4.21 min, >98% purity (254 nm). **HR-ESMS** calcd. for C_15_H_17_O_2_N_3_ [M+H]^+^: 272.1394; found 272.1398.

### 5.25. Ethyl 4-(4-Hydroxybut-1-yn-1-yl)pyrazolo[1,5-a]pyridine-3-carboxylate (***25***)

Ethyl 4-bromopyrazolo[1,5-*a*]pyridine-3-carboxylate (50 mg, 0.19 mmol) and but-3-yn-1-ol (39 mg, 0.56 mmol) were reacted using general procedure C to produce **25** as a brown powder (38 mg, 80%).

**^1^H NMR** δ 8.47 (d, *J* = 6.8 Hz, 1H), 8.39 (s, 1H), 7.50 (d, *J* = 7.2 Hz, 1H), 6.88 (t, *J* = 7.1 Hz, 1H), 4.33 (q, *J* = 7.1 Hz, 2H), 3.84 (t, *J* = 5.3 Hz, 2H), 2.73 (t, *J* = 5.3 Hz, 2H), 1.37 (t, *J* = 7.1 Hz, 3H). **^13^C NMR** δ 162.9 (C), 146.2 (CH), 140.0 (C), 133.1 (CH), 129.3 (CH), 115.6 (C), 113.5 (CH), 105.2 (C), 95.8 (C), 78.7 (C), 60.8 (CH_2_), 60.7 (CH_2_), 25.0 (CH_2_), 14.5 (CH_3_). **HPLC** *t*_R_ = 4.88 min, >99% purity (254 nm). **HR-ESMS** calcd. for C_14_H_14_O_3_N_2_ [M+H]^+^: 259.1077; found 259.1082.

### 5.26. Ethyl 4-(7-Hydroxyhept-1-yn-1-yl)pyrazolo[1,5-a]pyridine-3-carboxylate (***26***)

Ethyl 4-bromopyrazolo[1,5-*a*]pyridine-3-carboxylate (50 mg, 0.19 mmol) and hept-6-yn-1-ol (31 mg, 0.28 mmol) were reacted using general procedure C to produce **26** as a dark oil (39 mg, 70%).

**^1^H NMR** δ 8.43 (d, *J* = 6.9, 1H), 8.37 (s, 1H), 7.45 (d, *J* = 7.1 Hz, 1H), 6.84 (t, *J* = 7.1 Hz, 1H), 4.33 (q, *J* = 7.1 Hz, 2H), 3.65 (t, *J* = 6.1 Hz, 2H), 2.53 (t, *J* = 6.9 Hz, 2H), 1.99 (s, 1H), 1.70–1.51 (m, 6H), 1.37 (t, *J* = 7.1 Hz, 3H). **^13^C NMR** δ 162.5 (C), 146.0 (CH), 139.6 (C), 132.9 (CH), 128.8 (CH), 116.0 (C), 113.2 (CH), 105.7 (C), 98.2 (C), 77.1 (C), 62.7 (CH_2_), 60.2 (CH_2_), 32.3 (CH_2_), 28.1 (CH_2_), 25.2 (CH_2_), 20.0 (CH_2_), 14.6 (CH_3_). **HPLC** *t*_R_ = 5.61 min, >98% purity (254 nm). **HR-ESMS** calcd. for C_17_H_20_O_3_N_2_ [M+H]^+^: 301.1547; found 301.155.

### 5.27. Ethyl 4-(Cyclopropylethynyl)pyrazolo[1,5-a]pyridine-3-carboxylate (***27***)

Ethyl 4-bromopyrazolo[1,5-*a*]pyridine-3-carboxylate (50 mg, 0.19 mmol) and ethynylcyclopropane (18 mg, 0.28 mmol) were reacted using general procedure C to produce **27** as a dark green oil (31 mg, 66%).

**^1^H NMR** δ 8.42 (dd, *J* = 6.9, 0.9 Hz, 1H), 8.37 (s, 1H), 7.42 (dd, *J* = 7.2, 1.0 Hz, 1H), 6.82 (t, *J* = 7.1 Hz, 1H), 4.35 (q, *J* = 7.1 Hz, 2H), 1.53 (m, 1H), 1.39 (t, *J* = 7.1 Hz, 3H), 1.01–0.85 (m, 4H). **^13^C NMR** δ 162.4 (C), 146.0 (CH), 139.6 (C), 132.5 (CH), 128.8 (CH), 116.0 (C), 113.2 (CH), 105.8 (C), 101.6 (C), 72.2 (CH), 60.2 (CH_2_), 14.6 (CH_3_), 8.9 (2 × CH_2_), 0.8 (CH). **HPLC** *t*_R_ = 6.25 min, >97% purity (254 nm). **HR-ESMS** calcd. for C_15_H_14_O_2_N_2_ [M+H]^+^: 255.1128; found 255.1135.

### 5.28. Ethyl 4-(pent-1-yn-1-yl)pyrazolo[1,5-a]pyridine-3-carboxylate (***28***)

Ethyl 4-bromopyrazolo[1,5-*a*]pyridine-3-carboxylate (50 mg, 0.19 mmol) and pent-1-yne (19 mg, 0.28 mmol) were reacted using general procedure C to produce **28** as a yellow oil (22 mg, 46%).

**^1^H NMR** δ 8.45 (dd, *J* = 6.9, 0.8 Hz, 1H), 8.39 (s, 1H), 7.47 (d, *J* = 6.8 Hz, 1H), 6.85 (t, *J* = 7.1 Hz, 1H), 4.35 (q, *J* = 7.1 Hz, 2H), 2.50 (t, *J* = 7.1 Hz, 2H), 1.70 (sex, *J* = 7.3 Hz, 2H), 1.39 (t, *J* = 7.1 Hz, 3H), 1.06 (t, *J* = 7.4 Hz, 3H). **^13^C NMR** δ 162.5 (C), 146.1 (CH), 140.6 (C), 132.8 (CH), 129.0 (CH), 116.1 (C), 113.2 (CH), 98.4 (C), 78.7 (C), 77.1 (CH_2_), 60.7 (C), 60.2 (CH_2_), 22.1 (CH_2_), 14.6 (CH_3_), 13.8 (CH_3_). **HPLC** *t*_R_ = 6.58 min, >98% purity (254 nm). **HR-ESMS** calcd. for C_15_H_16_O_2_N_2_ [M+H]^+^: 257.1285; found 257.1284.

### 5.29. Ethyl 4-ethynylpyrazolo[1,5-a]pyridine-3-carboxylate (***29***)

Ethyl 4-((trimethylsilyl)ethynyl)pyrazolo[1,5-*a*]pyridine-3-carboxylate (30 mg, 0.10 mmol) in methanol (2 mL) was cooled to 0 °C; potassium carbonate (29 mg, 0.21 mmol) was added; then, the solution was stirred at room temperature for 5 h. Reaction mix was diluted with water (20 mL), extracted with ethyl acetate (25 mL), washed with brine (20 mL), dried over magnesium sulphate, filtered and concentrated in vacuo to produce **29** with sufficient purity as a brown oil (19 mg, 85%).

**^1^H NMR** δ 8.53 (d, *J* = 6.9 Hz, 1H), 8.44 (s, 1H), 7.62 (d, *J* = 7.2 Hz), 6.89 (t, *J* = 7.1 Hz, 1H), 4.37 (q, *J* = 7.1 Hz, 2H), 3.53 (s, 1H), 1.39 (t, *J* = 7.1 Hz, 3H). **^13^C NMR** δ 162.5 (C), 146.4 (CH), 139.2 (C), 134.8 (CH), 130.4 (CH), 114.2 (C), 113.0 (CH), 106.0 (C), 84.5 (C), 60.3 (CH_2_), 51.2 (CH), 14.9 (CH_3_). **HPLC** *t*_R_ = 5.08 min, >95% purity (254 nm). **HR-ESMS** calcd. for C_12_H_10_O_2_N_2_ [M+H]^+^: 215.0815; found 215.0814.

### 5.30. Surface Plasmon Resonance

SPR assays were performed on a Biacore S200 (GE Healthcare). His-tagged RT (WT, K103N, Y181C or K103N/Y181C) was immobilized on the surface of a Biacore compatible NIHC 1500M (Xantec) chip according to manufacturer’s instructions. Following 3 injections 300 s each of 0.5 M EDTA, the chip surface was activated with a 120 s injection of 5 mM NiCl_2_ followed by a 2100 s injection of 130 nM RT. The final immobilization level of RT was typically 14,000–27,000 RU. SPR experiments were carried out at 25 °C using a flow rate of 50 µL/min in running buffer. The running buffer was 1.053 × (HBS pH 7.4 + 50 µM EDTA + 0.005% Tween-20). Compounds were provided as 200 mM stocks and diluted in running buffer to obtain the concentrations required for dose-response studies (0, 6.25, 12.5, 25, 50, 100 and 200 µM). Raw sensorgram data were analyzed using Biacore S200 Evaluation Software. Dose-response determinations were evaluated using a 1:1 binding model. K_D_ values were determined by Biacore S200 Evaluation Software Version 1.0. assuming 1:1 binding. For compounds approaching saturation, Rmax was floated. For compounds with insufficient curvature, Rmax was fixed based on a control. Errors were determined as the standard error in the dose-response fit, as calculated by Biacore S200 Evaluation Software.

### 5.31. DNA Dependant DNA Polymerase (DDDP) Activity Assays

NNRTIs: Nevirapine was obtained from the AIDS Research and Reference Reagent Program, Division of AIDS, National Institute of Allergy and Infectious Diseases, NIH. Doravirine was purchased from MedChemExpress (Cat no HY-16767).

Recombinant HIV-1 RT: The p66/p51 HIV-1 RT heterodimer engineered with a N-terminal histidine tag was expressed from the pRT6H-PR vector (provided by Nicolas Sluis-Cremer) which is based on pRT6H-PROT [37], except that the RT coding region is derived from the infectious molecular clone xxLAI [38]. This xxLAI clone contains the HIV-1_LAI_ backbone [39,40] and is engineered with silent mutations introducing *Xma* I and *Xba* I sites in the RT coding region at nucleotide positions 40–45 and 1470 to 1475, respectively. pRT6H-PR expressing the NNRTI drug-resistant mutations K103N, Y181C and K103N/Y181C were generated by subcloning *Xma* I/*Xba* I fragments in xxLAI harboring the desired NNRTI mutations (Giacobbi NS and Sluis-Cremer 2017 AAC) into the corresponding restriction sites in the RT coding region of p6HRT-PR. RT was expressed in *Escherichia coli strain* M15 and purified using Ni-NTA chromatography as previously described [41,42]. RT concentrations were determined spectrophotometrically at 280 nm using an extinction coefficient (e_280_) of 260, 450 M^−1^ cm^−1^. To determine DDDP assay incubation times, RT stocks were subjected to a DDDP time course assay to determine the initial linear rate period, which was typically up to 10 min [14].

^33^P-radiolabeled DDDP assay: Compounds were initially screened for their ability to inhibit WT HIV-1 RT DDDP activity at 1000 µM, 333 µM and 111 µM. If at least 50% inhibitory activity was observed at any of the tested concentrations compared to the untreated control, the compounds were subsequently re-tested to determine their IC_50_ values against WT RT, with potent inhibitors tested against NNRTI drug-resistant mutants. Nevirapine was included in assays as a positive control for inhibiting RT polymerase activity. The DDDP assay was performed by first adding 25 ng of HIV-1 RT to test compound in 96-well plates followed by initiating RT polymerase activity by adding 200 ng of activated calf thymus (ACT) DNA (Sigma Aldrich), 0.2 µCi ^33^P-α-dTTP (Perkin Elmer), 50 mM Tris-HCl pH 7.8 50 mM KCl, 10 MgCl_2_, 50 µM dNTP mix (Bioline), 0.1 mg/mL bovine serum albumin (New England Biolabs), 0.01% IGEPAL (Sigma) and 1 mM dithiothreitol. Reactions contained 2.5% dimethyl sulfoxide (DMSO). Following incubation at 37 °C for 10 min, samples from duplicate wells were applied to Whatman DE81 ion-exchange paper (GE Healthcare); dried membranes were washed in 2X saline-sodium citrate (SSC) buffer; and incorporated counts were quantified using an FLP-2000 phosphorimager (Fujifilm) or Typhoon Trio Phosphorimager (GE Healthcare) as published [14]. Dose–response curves were generated using PRISM GraphPad version 8 by plotting the log transformed drug concentrations against percentage inhibition of DDDP activity compared to the no drug control using non-linear regression analysis [43].

Non-radioactive DDDP Assay: DDDP assays were also performed using a PicoGreen based spectrophotometric assay to detect double-stranded DNA synthesized by recombinant HIV-1 RT [15]. Assays were performed as described for the ^32^P-radiolabeled DDDP assay except that instead of ACT, the reaction contained 40 nM of a template/primer comprising a 200 nt DNA template (5′-TCTCTCTGGTTAGACCA GATCTGAGCCTGGGAGCTCTCTGGCTGACTAGGGAACCCACTGCTTAAGCCTCAATAAAGCTTGCCTTGAGTGCTTAAAGTAGATGTGTGTGCCCGTCTGTTGTGTGACTCTGGTAACTAGAGATCCCTCAGACCCTTTTAGTCAGTGTGGAATATCTCATAGCTTGGTGCTCGAACAGTGAC-3′) annealed to a 18 nucleotide DNA primer (5′-GTCACTGTTCGAGCACCA-3′), 50 mM Tris-HCl pH 7.8, 50 mM KCl, 10 mM MgCl_2_ and 50 µM dNTP mix. Reactions were stopped by adding the detection reagent containing 1 × Quant-IT PicoGreen Reagent (Invitrogen) and 12.5 mM ethylenediaminetetraacetic acid (EDTA); samples were transferred to a 96-well black plate, and fluorescence was measured at excitation/emission (480/520 nm) using the CLARIOstar multifunction microplate reader (BMG Labtech).

Combination Assays: To determine if compounds mediated synergistic, antagonistic or additive inhibition of recombinant HIV-1 RT, the non-radioactive DDDP assay was used as described above except that the template/primer comprised an 18 nt primer annealed to a 100 nt template (5′-ATGTGTGTGCCCGTCTGTTGTGTGACTCTGGTAAC TAGAGATCCCTCAGACCCTTTTAGTCAGTGTGGAATATCTCATAGCTTGGTGCTCGAACAGTGAC-3′) [15], and the Quantifluor dsDNA system (Promega) was used, according to the manufacturer’s protocol, instead of the Quant-IT PicoGreen Reagent. The combination analysis performed was based on the median effect principle (Chou and Talalay 1983). Data were analyzed using the CalcuSyn Version 2 software (Biosoft, Cambridge, UK) according to CalcuSyn user manual instructions for fixed-ratio combinations [44]. Nevirapine (NVP) was tested in combination with doravirine (DOR), compound **B**-**1** and **27** at fixed ratios of 18:1, 1:200 and 1:19, respectively. Combination index (CI) values at effective doses of 50% (ED_50_), 75% (ED_75_) and 90% (ED_90_) were calculated from three independent assays demonstrating a median-effect plot linear correlation coefficient (*r*) of *r* > 0.96. Average CI and the SEM was calculated for 3 independent assays. CI 0.90–1.10 is nearly additive; CI 1.10–1.20 is slight antagonism; CI 1.20–1.45 is moderate antagonism.

### 5.32. HIV-1 RT Crystallization and Structure Determination

RT52A, a crystallization-optimized form of RT, was expressed and purified as previously described [45]. Hanging-drop crystallization trays were set up following incubation of RT52A (20 mg/mL) for 1 h with either 1:12 **27** or 1:12 **27** plus 1:1.5 rilpivirine (RPV) molar ratio at room temperature (~23 °C). Crystals were grown at 4 °C in either 1:2, 1:1 or 2:1 ratios of protein and well solution containing 11% PEG 8000, 4% PEG 400, 100 mM imidazole pH 6.6, 10 mM spermine, 15 mM MgSO_4_, 100 mM ammonium sulfate and 5 mM tris(2-carboxyethyl)phosphine (TCEP) and a previously optimized concentration of microseeds from RT52A/RPV crystals.

Cryoprotectant solutions were prepared using crystallization well solution with the addition of 5% (*v*/*v*) ethylene glycol, and 20% (*v*/*v*) DMSO (containing 2 mM final concentration of **27** or 0.256 mM rilpivirine plus 2 mM **27**). Crystals were harvested ~3 weeks after appearance. Crystals were placed in cryoprotectant for 5 min before flash-cooling in liquid nitrogen. Data were collected at NSLS-II AMX and APS 23ID-D beamlines. Diffraction data were indexed, processed, scaled and merged using the fastDP (XDS) pipeline [46]. Merged datasets were passed through CCP4 DIMPLE to assess ligand binding [47]. Unmerged datasets were assessed for isomorphism using CCP4 BLEND [48], in analysis mode. Three datasets—whose Linear Cell Variation (LCV) values were <1.5%—were merged using CCP4 BLEND Synthesis and AIMLESS [49]. Ligand coordinates and restraints were generated with the Grade Web Server [50]. Molecular replacement, model building and refinement were performed using Phaser (with PDB ID 4G1Q as template) [51], Coot [52] and phenix.refine [53], respectively. The diffraction data and refinement statistics are summarized in Table S1. Figures were prepared with PyMOL (The PyMOL Molecular Graphics System, Version 2.5 Schrödinger, LLC, New York, NY, USA) and BioRender (created with BioRender.com, Toronto, ON, Canada).

Statistical Analysis: The statistical significance between IC_50_ values obtained from recombinant RT assays were determined using Mann–Whitney test or Kruskal–Wallis test for multiple comparisons using Prism 9 (GraphPad Software Version 9.4.0, Boston, MA, USA).

## Figures and Tables

**Table 1:** HIV-1 RT inhibition, dissociation constants and ligand efficiencies of substituted ethyl 3-carboxypyrazolopyridine analogues (Series 1).

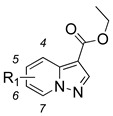								
#	R_1_	RT Inhibition—IC_50_ ± SEM (µM) ^a^			SPR—K_D_ (µM) ^b^			LE ^c^
		WT	K103N	Y181C	WT	K103N	Y181C	
**B-1**	-	>1000 ^d^	-	-	NB	NB	NB	-
**2**	4-CH_3_	433 ± 166	-	>333	347 ± 23	-	-	0.31
**3**	5-CH_3_	>1000	-	-	-	-	-	-
**4**	6-CH_3_	>1000	-	-	-	-	-	-
**5**	7-CH_3_	470 ± 95	-	>333	NB	NB	NB	0.31
**6**	4-Br	159 ± 29	846 ± 42	850 ± 187	598 ± 15	1400 ± 45	1190 ± 47	0.35
**7**	5-Br	>1000	-	-	-	-	-	-
**8**	5-NH_2_	>1000	-	-	522 ± 27	621 ± 36	610 ± 32	-
**9**	4-Br, 7-CH_3_	155 ± 19 ^d^	-	-	NB	NB	-	0.33
**10**	4-Br, 6-OCH_3_	315 ± 19 ^d^	-	-	1570 ± 105	757 ± 81	-	0.29
**11**	4-F, 7-CH_3_	>1000 ^d^	-	-	NB	NB	-	-
**12**	4-Cl, 7-CH_3_	>1000 ^d^	-	-	-	-	-	-
**13**	4-OCH_3_, 6-Br	>1000	-	-	-	-	-	-
**14**	5-Cl, 7-CH_3_	>1000	-	-	NB	NB	-	-
**15**	5-COOCH_3_	>1000	-	-	-	-	-	-
**16**	5-NHCOOC(CH_3_)_3_	>1000	-	-	-	-	-	-

^a^ The 50% inhibitory concentration (IC_50_) values ± SEM (standard error of the mean) were determined by assessing inhibition of HIV-1 RT DNA-dependent DNA Polymerase (DDDP) activity using the nonradioactive PicoGreen or ^33^P radiolabeled assay. IC_50_ values were determined from at least *n* ≥ 2 independent assays. ^b^ Dissociation constants (K_D_) ± standard error in the dose–response fit, as calculated by Biacore S200 Evaluation Software] (*n* = 1 independent assays) were measured using surface plasmon resonance (SPR). ^c^ Ligand efficiency (LE) is expressed as kcal/mol/HA where HA represents the number of non-hydrogen atoms in the compound. ^d^ RT DDDP inhibitory activity determined using the PicoGreen assay. NB denotes no binding observed.—denotes not determined.

**Table 2:** HIV-1 RT inhibition, dissociation constants and ligand efficiencies of substituted 4-alkyne pyrazolopyridine analogues (Series 3).

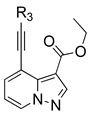						
#	R_3_	RT Inhibition—IC_50_ ± SEM (µM) ^a^		SPR—*K_D_* (µM) ^b^		LE ^c^
		WT	K103N/Y181C	WT	K103N	
**24**	CH_2_N(CH_3_)_2_	>1000	-	-	-	-
**25**	CH_2_CH_2_OH	>1000	-	-	-	-
**26**	CH_2_(CH_2_)_4_OH	24 ± 6	35 ± 7 ^d^	173 ± 9	233 ± 22	0.30
**27**	cyclopropyl	11 ± 1	34 ± 8 ^d^	79 ± 7	187 ± 15	0.37
**28**	CH_2_CH_2_CH_3_	53 ± 4	105 ± 6 ^d^	275 ± 9	-	0.32
**29**	H	27 ± 26	43 ± 14 ^d^	395 ± 35	-	0.40

^a^ The 50% inhibitory concentration (IC_50_) values ± SEM (standard error of the mean) were determined by assessing inhibition of HIV-1 RT DNA-dependent DNA Polymerase (DDDP) activity using the nonradioactive PicoGreen or ^33^P radiolabeled assay. IC_50_ values were determined from at least *n* ≥ 2 independent assays. ^b^ Dissociation constants (K_D_) ± standard error in the dose–response fit, as calculated by Biacore S200 Evaluation Software] (*n* = 1 independent assay) were measured using surface plasmon resonance (SPR). ^c^ Ligand efficiency (LE) is expressed as kcal/mol/HA where HA represents the number of non-hydrogen atoms in the compound. ^d^ RT DDDP inhibitory activity determined using the PicoGreen assay.—denotes not determined.

**Table 3:** Compounds **B-1** and **27** in combination with nevirapine antagonistically inhibit HIV-1 RT.

Drug Combinations ^a^	CI ED_50_ ± SEM ^b^	CI ED_75_ ± SEM	CI ED_90_ ± SEM	Description
NVP vs. DOR ^c^	0.95 ± 0.09	0.86 ± 0.16	0.92 ± 0.27	Nearly additive
NVP vs. **B**-**1**	1.28 ± 0.04	1.38 ± 0.11	1.54 ± 0.26	Moderate antagonism
NVP vs. **27**	1.28 ± 0.03	1.42 ± 0.15	1.64 ± 0.35	Moderate antagonism

^a^ Drugs tested in combination to determine synergistic, additive, or antagonistic inhibition of HIV-1 RT. ^b^ Combination index (CI) at the effective doses of 50% (ED_50_), 75% (ED_75_) or 90% (ED_90_) for inhibition of HIV-1 RT activity using the nonradioactive DDDP assay. SEM denotes standard error of the mean of CI values from *n* = 3 independent assays. CI 0.90–1.10 is nearly additive; CI 1.10–1.20 is slight antagonism; CI 1.20–1.45 is moderate antagonism. ^c^ Assays for NVP (nevirapine) and DOR (doravirine) were performed using a 100 nt/18 nt DNA template/primer and QuantiFluor in contrast to the other drug combinations which were formed with a 200 nt/18 nt DNA template/primer and the Quant-IT PicoGreen reagent.

**Table 4:** PyVOL pocket volume analysis of the NNIBP and NNRTI Adjacent site in NNRTI-bound RT structures. A minimum and maximum probe radius of 1.8 and 3.4 Å was used. Dashes indicate a closed pocket with no measurable volume.

PDB ID	Ligand	Pocket Volume (Å^3^)	
		NNRTI Adjacent Site	NNIBP
1IKW	Efavirenz	-	398
4NCG	Doravirine	-	426
3MEC	Etravirine	-	436
8FFX	**27**	-	436
1KLM	Delavirdine	-	446
1VRT	Nevirapine	-	455
4G1Q	Rilpivirine	416	512
4KFB	Rilpivirine/compound **B**-**1**	413	527
7KWU	K07-15	597	676
6C0J	K-5a2	542	685
6C0N	25a	479	687

## Data Availability

Data and protocols associated with this publication not included in the manuscript and supplementary can be made available upon request. The RT/compound **27** structure has been deposited in the Protein Data Bank (PDB ID 8FFX). Atomic coordinates and experimental data will be released upon article publication.

## Supplementary Materials

The following supporting information can be downloaded at: https://www.mdpi.com/article/10.3390/molecules28073103/s1, Table S1: Binding site, chemical structure, and potency of HIV-1 RT fragments hits discovered by Bauman et al.; Table S2: HIV-1 RT inhibition, dissociation constants and ligand efficiencies of Series 2 compounds; Figure S1: SPR curves for compounds **2**, **6** and **27**; Figure S2: Dose response curves for compounds **B-1** and **27** determined using the Picogreen RT DDDP assay; Table S3: X-ray data and refinement statistics for PDB 8FFX; Figure S3: Interactions of compound **27** with neighboring waters in the NNIBP; Figure S4: SWISSADME analysis summary of compound **27**; Figure S5: Chemical structures of NNRTIs and compound **27** described in this study.
