# Supplementary material for: Targeting HIV-1 Reverse Transcriptase Using a Fragment-Based Approach
Source: Molecules. 2023 Mar 30;28(7):3103. doi: 10.3390/molecules28073103 (PMC10095864; doi:10.3390/molecules28073103)
Supplement: Supplementary file 1 [file molecules-28-03103-s001.zip › molecules-2156981-supplementary.pdf]

<u>**Supporting Information**</u>

Targeting HIV-1 Reverse Transcriptase using a Fragment-Based Approach

Mahta Mansouri [1,†], Shawn Rumrill [2,†], Shane Dawson [1], Adam Johnson [3,] Jo-Anne Pinson [1], Menachem J. Gunzburg [1], Catherine F. Latham [3], Nicholas Barlow [1,] George W. Mbogo [3], Paula Ellenberg [3], Stephen J. Headey [1], Nicolas Sluis-Cremer [4,] David Tyssen [3], Joseph D. Bauman [2], Francesc X. Ruiz [2], Eddy Arnold [2,*], David K. Chalmers [1,*] and Gilda Tachedjian [3,5,6,*]

[1] Medicinal Chemistry, Monash Institute of Pharmaceutical Sciences, Monash University, Parkville, VIC 3052, Australia
[2] Center for Advanced Biotechnology and Medicine, and Department of Chemistry and Chemical Biology, Rutgers University, Piscataway, NJ 08854, USA
[3] Retroviral Biology and Antivirals Laboratory, Disease Elimination Program, Life Sciences Discipline, Burnet Institute, Melbourne, VIC 3004, Australia
[4] Division of Infectious Diseases, Department of Medicine, University of Pittsburgh School of Medicine, Pittsburgh, PA 15261, USA
[5] Department of Microbiology, Monash University, Clayton, VIC 3168, Australia
[6] Department of Microbiology and Immunology at Peter Doherty Institute for Infection and Immunity, University of Melbourne, Melbourne, VIC 3000, Australia
* Correspondence: arnold@cabm.rutgers.edu (E.A.); david.chalmers@monash.edu (D.K.C.); gilda.tachedjian@burnet.edu.au (G.T.)
† These authors contributed equally to this work.

Supporting information table of contents

1. Table S1: Binding site, chemical structure, and potency of HIV-1 RT fragments hits discovered by Bauman *et al.*
2. Table S2: HIV-1 RT inhibition, dissociation constants and ligand efficiencies of Series 2 compounds
3. Figure S1: SPR curves for compounds **2**, **6** and **27**
4. Figure S2: Dose response curves for compounds **B-1** and 27 determined using the Picogreen RT DDDP assay
5. Table S3: X-ray data and refinement statistics for PDB 8FFX
6. Figure S3: Interactions of compound **27** with neighboring waters in the NNIBP
7. Figure S4: SWISSADME analysis summary of compound **27**
8. Figure S5: Chemical structures of NNRTIs and compound **27** described in this study

Table S1. Binding site, chemical structure, and potency of HIV-1 RT fragments hits discovered by Bauman et al. [7].

Site	IC_{50} (μM)	Compound	Structure
428	N/A	7	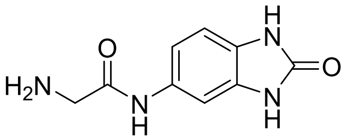
RNase H Primer Grip Adjacent	N/A	9	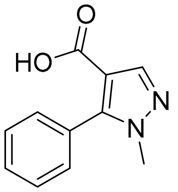
507	150	8	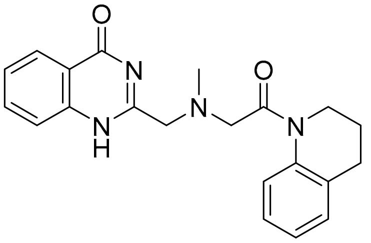
399	N/A	6	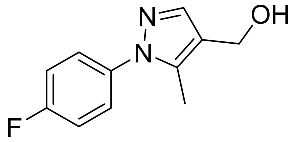
NNRTI Adjacent	350	**B-1**	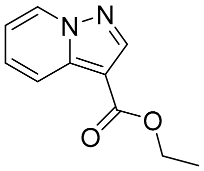
Knuckles	600	3	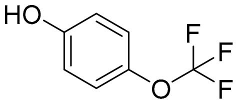
Incoming Nucleotide Binding	200	5	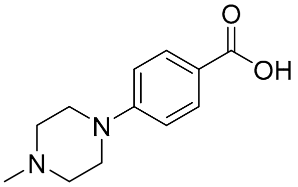

Table S2. HIV-1 RT inhibition, dissociation constants and ligand efficiencies of Series 2 compounds.

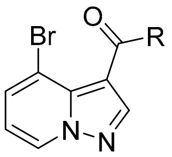

#	R1	RT Inhibition - IC_{50} ± SEM (μM)[a]	SPR - K_D (μM)[b]		
		WT	WT	K103N	Y181C
**17**	-OH	>1000	NB	NB	NB
**18**	-OCH3	>1000[c]	-	-	-
**19**	$-OCH_2CH_3$	>1000[c]	-	-	-
**20**	$-NHCH_2CH_3$	>1000	-	-	-
**21**	$-N(CH_2CH_3)_2$	-	-	-	-
**22**	$-NHCH_2CH_2PhOH$	111.4[c]	-	-	-

[a] The 50% inhibitory concentration (IC_{50}) values were determined by assessing inhibition of HIV-1 RT DNA-dependent DNA Polymerase (DDDP) activity using the nonradioactive PicoGreen or ^{33}P radiolabeled assay. IC_{50} values were determined from at least $n \geq 2$ independent assays. [b]Dissociation constants (K_D) were measured using surface plasmon resonance (SPR). [c]RT DDDP inhibitory activity determined using the PicoGreen assay. NB denotes no binding observed. – denotes not determined.

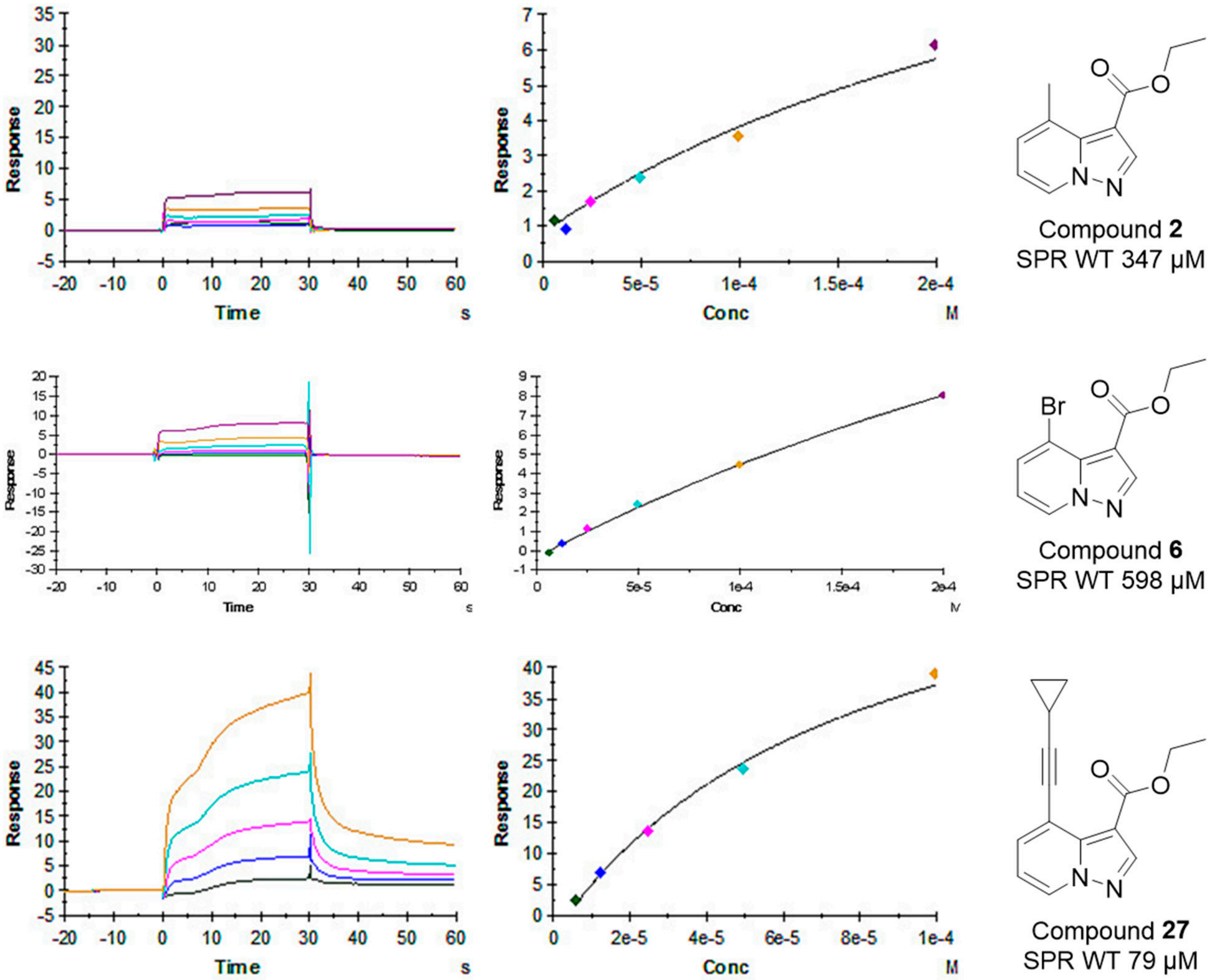

Figure S1. SPR curves for compounds **2**, **6** and **27.**

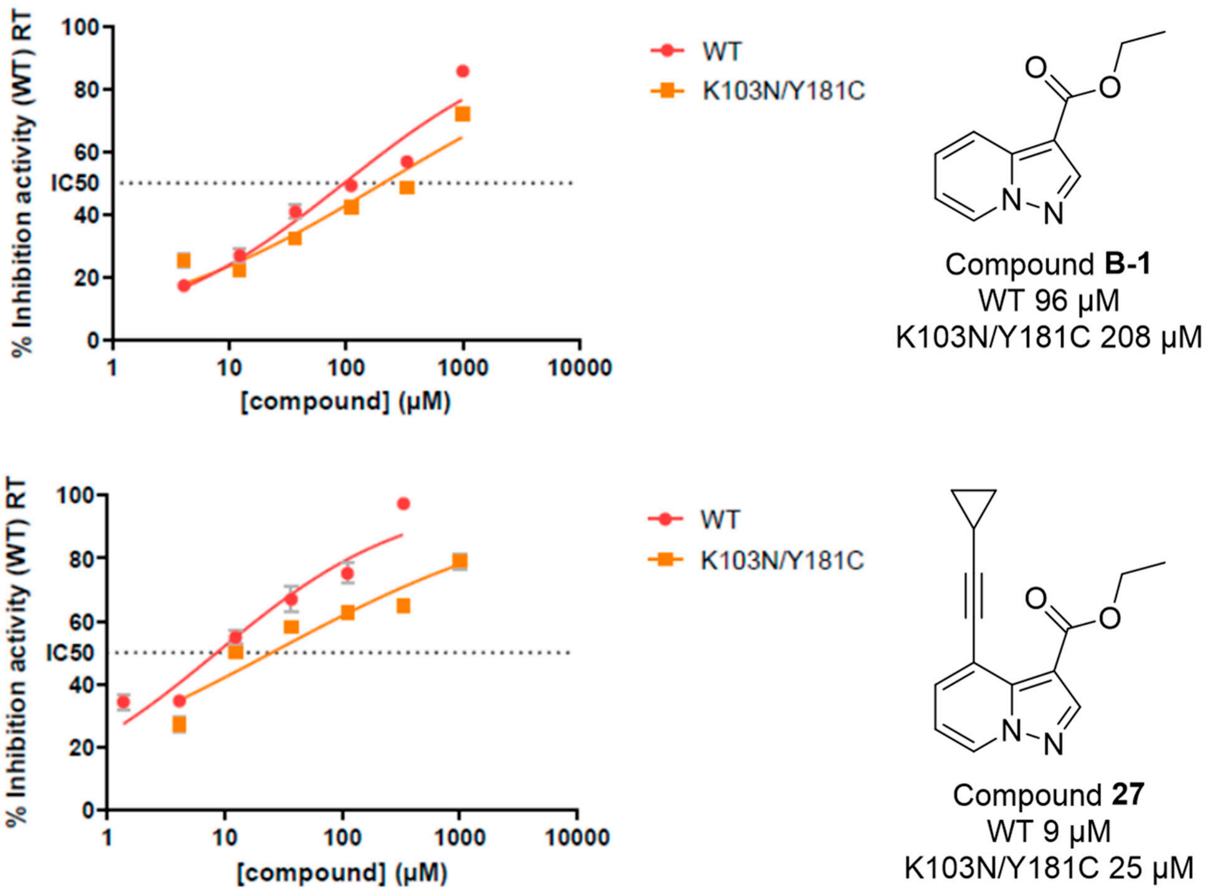

Figure S2. Dose response curves for compounds **B-1** and **27** determined using the Picogreen RT DDDP assay.

Table S3. X-ray data and refinement statistics for PDB 8FFX.

Protein Data Bank (PDB) accession code	8FFX
Wavelength (Å)	1.033
Resolution range (last shell) (Å)	48.29 - 2.42 (2.51 - 2.42)
Space group	C2
Cell constants (a, b, c in Å; α, β, γ in °)	161.95, 73.38, 107.97, 90.0, 100.1, 90.0
Total reflections (last shell)	95241 (9331)
Unique reflections (last shell)	47666 (4692)
Multiplicity (last shell)	2.0 (2.0)
Completeness (last shell) (%)	98.98 (95.54)
Mean I/sigma(I) (last shell)	9.75 (1.62)
Wilson B-factor	54.47
R-merge (last shell)	0.088 (-17.150)
R-meas	0.124 (-24.260)
R-pim	0.088 (-17.150)
CC1/2	0.425 (0.288)
CC*	0.772 (0.668)
Reflections used in refinement	47358 (4556)
Reflections used for R-free	1989 (193)
R-work	0.214 (0.335)
R-free	0.266 (0.394)
CC(work)	0.951 (0.374)
CC(free)	0.923 (0.549)
Number of non-hydrogen atoms	8124
macromolecules	7951
ligands	40
solvent	123
Protein residues	971

Nucleic acid bases	N/A
RMS(bonds)	0.005
RMS(angles)	0.70
Ramachandran favored (%)	96.06
Ramachandran allowed (%)	3.73
Ramachandran outliers (%)	0.23
Rotamer outliers (%)	0.46
Clashscore	6.79
Average B-factor	82.23
macromolecules	82.54
ligands	91.00
solvent	58.49
Number of TLS groups	1

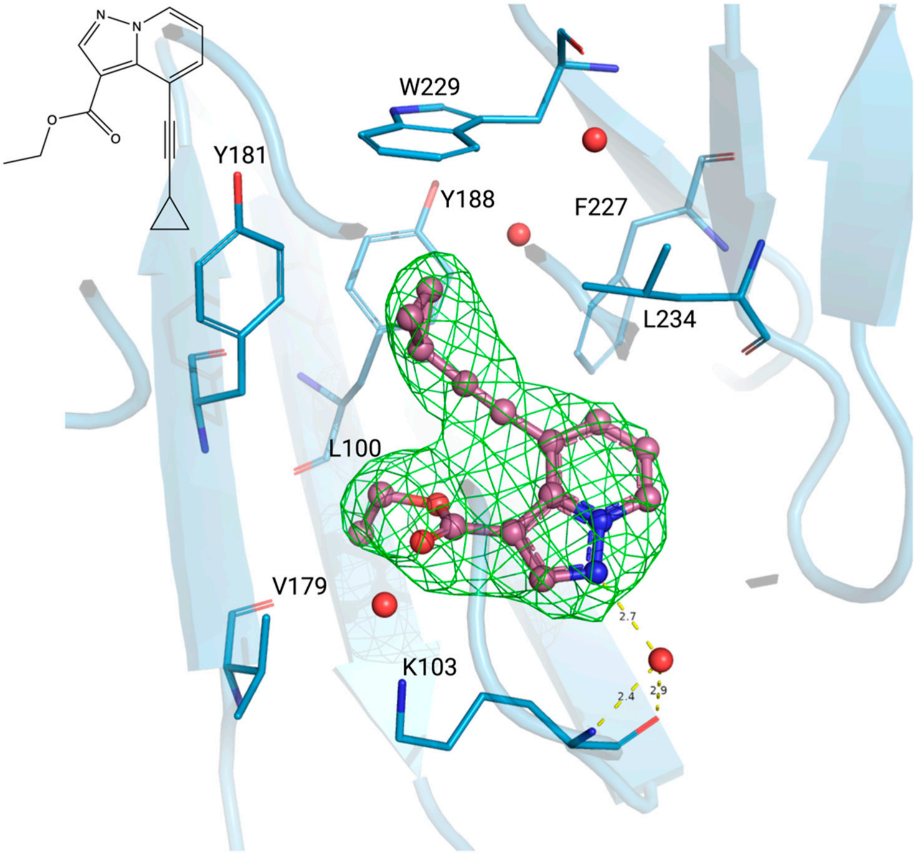

Figure S3. Interactions of compound **27** with neighboring waters in the NNIBP (PDB ID 8FFX). Atomic model of **27** (white) bound to HIV-1 RT residues (pale cyan). Pocket residues forming hydrophobic interactions shows as sticks. Polder OMIT mFo–DFc map density (green mesh, 3σ) of **27**. Water molecules shown as red spheres. Created using Pymol and BioRender.com.

Molecule 1

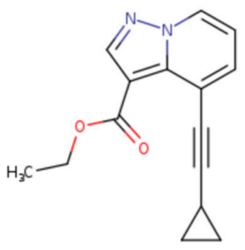

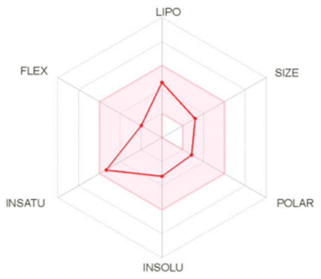

SMILES CCOC(=O)c1cnn2c1c(ccc2)C#CC1CC1

Physicochemical Properties	
Formula	C15H14N2O2
Molecular weight	254.28 g/mol
Num. heavy atoms	19
Num. arom. heavy atoms	9
Fraction Csp3	0.33
Num. rotatable bonds	3
Num. H-bond acceptors	3
Num. H-bond donors	0
Molar Refractivity	71.52
TPSA	43.60 Å^2

Lipophilicity	
Log $P_{o/w}$ (iLOGP)	2.76
Log $P_{o/w}$ (XLOGP3)	2.59
Log $P_{o/w}$ (WLOGP)	2.29
Log $P_{o/w}$ (MLOGP)	2.89
Log $P_{o/w}$ (SILICOS-IT)	2.50
Consensus Log $P_{o/w}$	2.61

Water Solubility	
Log *S* (ESOL)	-3.20
Solubility	1.60e-01 mg/ml ; 6.30e-04 mol/l
Class	Soluble
Log *S* (Ali)	-3.15
Solubility	1.78e-01 mg/ml ; 7.01e-04 mol/l
Class	Soluble
Log *S* (SILICOS-IT)	-3.25
Solubility	1.42e-01 mg/ml ; 5.57e-04 mol/l
Class	Soluble

Pharmacokinetics	
GI absorption	High
BBB permeant	Yes
P-gp substrate	No
CYP1A2 inhibitor	Yes
CYP2C19 inhibitor	Yes
CYP2C9 inhibitor	Yes
CYP2D6 inhibitor	No
CYP3A4 inhibitor	No
Log K_p (skin permeation)	-6.01 cm/s

Druglikeness	
Lipinski	Yes; 0 violation
Ghose	Yes
Veber	Yes
Egan	Yes
Muegge	Yes
Bioavailability Score	0.55

Medicinal Chemistry	
PAINS	0 alert
Brenk	1 alert: triple_bond
Leadlikeness	Yes
Synthetic accessibility	2.66

Figure S4. SWISSADME analysis (http://www.swissadme.ch/) summary of compound **27**.

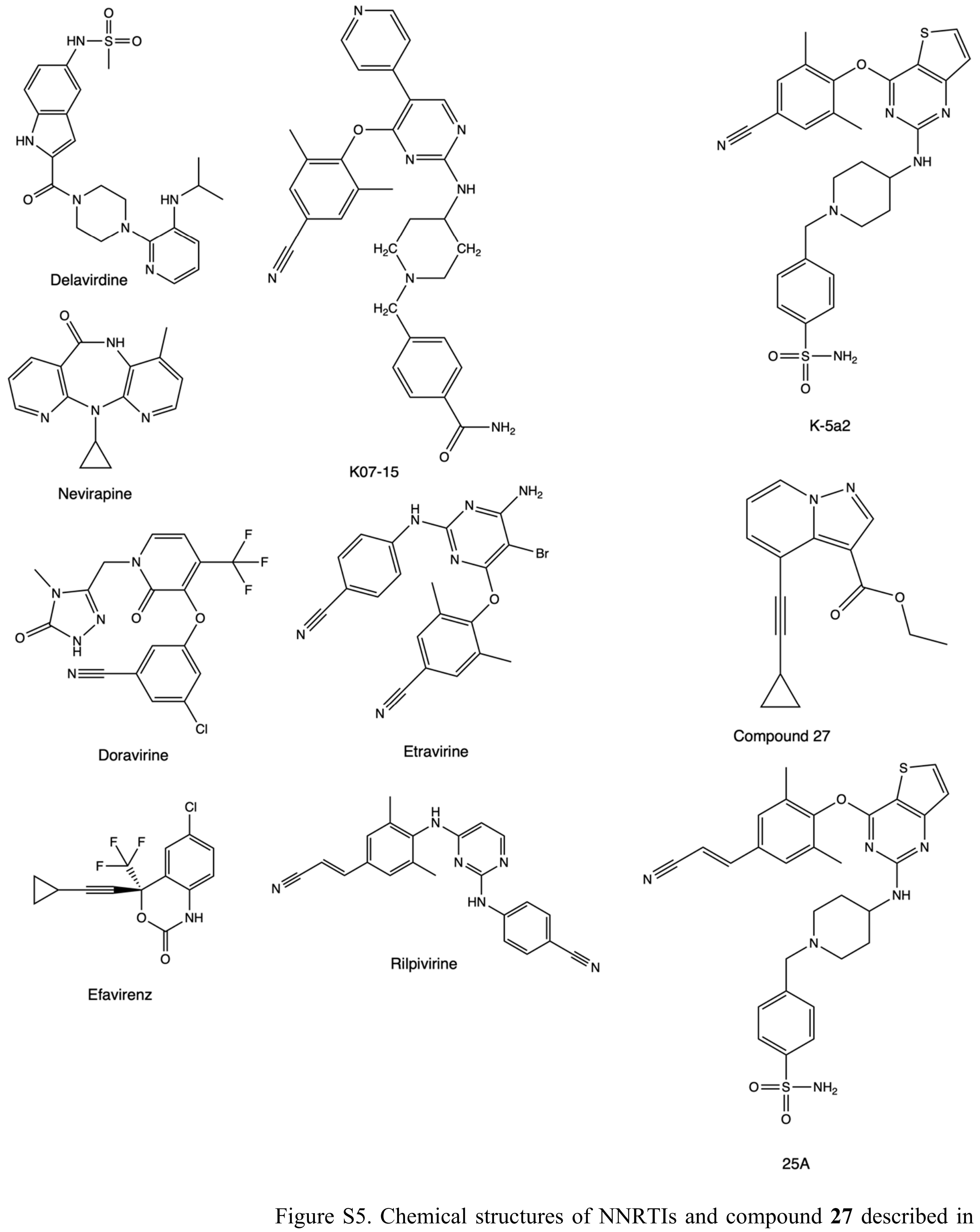

Figure S5. Chemical structures of NNRTIs and compound **27** described in this study.
